# Removal of Hexavalent Chromium from Water Using Cork-Based Biochars Obtained via Slow and Microwave Pyrolysis

**DOI:** 10.3390/molecules30234501

**Published:** 2025-11-21

**Authors:** Eva Pertile, Tomáš Dvorský, Vojtěch Václavík, Lucie Berkyová, Petr Balvín

**Affiliations:** Department of Environmental Engineering, Faculty of Mining and Geology, VSB—Technical University of Ostrava, 17. Listopadu 15/2172, 708 00 Ostrava, Czech Republic; eva.pertile@vsb.cz (E.P.); vojtech.vaclavik@vsb.cz (V.V.);

**Keywords:** biochar, microwave pyrolysis, slow pyrolysis, cork, hexavalent chromium, adsorption, biomass waste, pH effect, adsorption isotherms, adsorption kinetics

## Abstract

Cork-derived biochars produced by slow pyrolysis (C-SP) and microwave-assisted pyrolysis (C-MWP) were evaluated for the removal of hexavalent chromium [Cr(VI)] from aqueous solutions. The materials were characterized by proximate/elemental analysis, N_2_ physisorption (BET), thermogravimetry and FTIR. C-SP exhibited a higher specific surface area (174.5 m^2^·g^−1^), a larger micropore volume and stronger O–H/C–O surface signatures than C-MWP, which showed partial aromatization and less-developed texture. Batch equilibrium adsorption experiments at pH 2.0 with initial Cr(VI) concentrations of 5–150 mg·L^−1^ and kinetic experiments (*c*_0_ = 40 mg·L^−1^) were analyzed by nonlinear fitting to standard isotherm and kinetic models. The experimentally observed maximum uptakes were *qmax*,*obs* = 32.7 mg·g^−1^ (C-SP) and 35.9 mg·g^−1^ (C-MWP) at pH 2.0. Under screening conditions (pH 1.1, 45 min), removals reached 72% (1.44 mg·g^−1^) for C-SP and 87% (1.73 mg·g^−1^) for C-MWP. The Freundlich model provided the best fit to the equilibrium data for C-SP, consistent with adsorption on an energetically heterogeneous surface, whereas for C-MWP the fitted isotherm models did not yield physically meaningful parameters. Kinetic modeling showed rapid initial uptake consistent with energetic heterogeneity of surface sites. However, kinetic fits alone do not determine whether uptake is dominated by physical adsorption, chemical binding or redox reactions; direct surface/speciation analyses would be required to substantiate mechanistic assignments.

## 1. Introduction

Hexavalent chromium [Cr(VI)] represents a highly toxic and carcinogenic form of chromium that threatens human health and aquatic ecosystems even at very low concentrations. Compared to trivalent chromium [Cr(III)], Cr(VI) is more soluble in water, more mobile, and more bioavailable [1,2]. It readily penetrates cell membranes via the sulfate transporters, where it undergoes intracellular reduction accompanied by the formation of reactive oxygen species, thereby increasing its genotoxic potential. Exposure to Cr(VI) can result in DNA (deoxyribonucleic acid) damage as well as respiratory and gastrointestinal disorders, which is why its occurrence in drinking and industrial waters is strictly regulated worldwide [1,2,3].

Anthropogenic sources of Cr(VI) include electroplating, leather tanning, stainless steel production, textile manufacturing, chromate synthesis, and particularly the improper disposal of industrial wastes. Historical contamination from mining and industrial activities further contributes to elevated Cr levels in surface and groundwater. Conventional remediation methods such as chemical reduction/precipitation, ion exchange, membrane filtration, and electrochemical treatments are effective but often suffer from high operational costs, energy demands, and the generation of secondary waste streams [2].

Adsorption using low-cost sorbents has therefore emerged as a widely investigated and practical approach for Cr(VI) remediation due to its operational simplicity and low capital cost compared with many physicochemical treatment methods. Recent reviews summarize extensive laboratory-scale work on biochar-based removal of Cr(VI), including both unmodified and chemically modified materials [4]. Reported adsorption capacities of lignocellulosic biochars typically range from 20 to 120 mg·g^−1^, depending on the feedstock type, pyrolysis temperature, and surface modification method, which places the present study within the performance range observed for similar materials. Representative mechanisms reported for Cr(VI) removal by biochar and related carbons under acidic conditions include: (i) electrostatic attraction of HCrO_4_^−^ to protonated surface sites, (ii) specific complexation at oxygenated functionalities, and (iii) adsorption-coupled reduction of Cr(VI) to Cr(III) on redox-active moieties, with Cr(III) detected on spent sorbents by surface-sensitive techniques. These pathways have been documented across unmodified and modified carbons and are consistent with the broader biochar literature on Cr(VI) remediation [5,6,7,8,9]. Because surface functionality and texture jointly govern these pathways, feedstock type and pyrolysis modality (SP vs. MWP) are expected to modulate uptake behavior. In recent years, biochar (a carbon-rich material produced by pyrolyzing biomass under oxygen-limited conditions) has gained attention for its porous structure, surface functionality, and tunable physicochemical properties. Owing to these features, biochar not only adsorbs Cr(VI) but may also facilitate its reduction to the less toxic Cr(III) [3].

However, sorption is just one of several advanced technologies explored for heavy metal remediation. Photocatalysis, particularly using TiO_2_-based composites, enables solar-driven reduction of Cr(VI) with high efficiency under mild conditions [10,11]. Membrane filtration offers precise separation but is limited by fouling and cost [12]. Capacitive deionization (CDI), an emerging electrochemical method, allows selective ion removal with low energy input, though its application to Cr(VI) is still under development [13]. Cloud point extraction (CPE) and nanomaterial-based adsorbents have also shown promise, especially for trace-level removal and simultaneous detection [14,15]. Comparative studies reveal that while photocatalysis and CDI offer high removal efficiencies and potential for regeneration, their scalability and long-term stability remain challenges. Adsorption using biochar stands out for its simplicity, low cost, and adaptability to various water matrices. Moreover, biochar can be modified (e.g., magnetically functionalized or composited with photocatalysts) to enhance performance [16,17].

The effectiveness of biochar strongly depends on the feedstock type and pyrolysis conditions [18]. SP typically yields biochars with stable carbon structures and well-developed porosity, whereas MWP enables rapid heating, which may produce materials with distinct surface chemistry and energy profiles [18,19,20]. Despite the growing body of literature on Cr(VI) removal using biochar, systematic comparisons between SP and MWP for the same feedstock are scarce.

Cork biomass (*Quercus suber*), abundant in Mediterranean regions, is a lignocellulosic material with low ash content and a cellular structure favorable for conversion into porous carbonaceous adsorbents. Cork-derived biochars have demonstrated high efficiency in removing heavy metals, including Cr(VI), especially when modified with magnetic or photocatalytic components [21,22,23,24].

This study’s novelty lies in a head-to-head comparison of slow- and microwave-pyrolyzed cork biochars prepared from the same feedstock, evaluated under matched acidic conditions with combined kinetic and equilibrium analyses. Beyond standard reporting, we estimate apparent active-site densities (from *qmax*,*obs* and *S_BET_*) to contextualize uptake beyond total surface area. The materials are thoroughly characterized (proximate/elemental analysis, N_2_ physisorption/BET, thermogravimetry, FTIR) and evaluated for Cr(VI) removal via integrated kinetic and equilibrium tests. To place the results in context, we benchmark observed uptakes against literature values for cork- and other biomass-derived biochars (see Supplementary Materials, Table S1) and compute site-density estimates (Table S2). Together, these data clarify how pyrolysis modality tunes textural and surface-chemical features and thereby modulates Cr(VI) uptake for an understudied feedstock. We also state the study’s limitations: routine surface/speciation analyses (e.g., XPS, pHpzc, or total-Cr/Cr(VI) mass balances) were not performed here and would be required to unambiguously discriminate adsorption from surface-mediated redox processes in future work.

Despite the rapidly growing number of studies on Cr(VI) remediation using biochar, systematic like-for-like comparisons of SP vs. MWP on the same feedstock remain scarce. While SP follows a controlled thermal regime and MWP a power-driven heating mechanism, here we restrict our comparison to texture/surface characteristics and adsorption performance under matched acidic conditions. Techno-economic and energy assessments are beyond the scope of this bench-scale study and are noted as directions for future work.

## 2. Results and Discussion

### 2.1. Characterization of Cork-Derived Biochars

Cork biochars produced by SP and MWP were characterized and compared using a property-centered approach rather than by nominal set-point temperatures. SP and MWP employ fundamentally different heating mechanisms: SP follows a controlled external temperature program, while MWP is governed by applied microwave (MW) power and therefore typically produces different heating rates, localized hot spots, and thermal variation that cannot be equated directly to a single furnace temperature. Such behavior of MW heating has been previously described and quantified in the literature [25] and motivated our property-centered comparative strategy.

Accordingly, both char types were subjected to the same suite of quantitative analyses on the final, stabilized products: proximate and ultimate elemental composition, char yield, ash and fixed-carbon content, thermal decomposition behavior (TGA/DTG), textural properties (N_2_-BET surface area, total/micro/mesopore volumes) and surface functional groups (FTIR).

This property-centered presentation focuses on parameters that directly determine adsorption behavior (surface area and pore architecture, surface functional groups, chemical composition, and thermal stability), enabling attribution of observed differences in Cr(VI) uptake to genuine physicochemical consequences of the two pyrolytic pathways rather than to artifacts arising from non-equivalent temperature control.

#### 2.1.1. Proximate and Elemental Analysis

Raw cork biomass (C-RAW) contained ~79 wt.% volatiles, ~2.6 wt.% ash, and 15 wt.% fixed carbon, which is consistent with reported values for lignocellulosic materials enriched in suberin. After pyrolysis, both SP and MWP processing resulted in pronounced removal of volatile matter (to ~12 wt.% respectively) and a corresponding enrichment of fixed carbon (>83 wt.%). The ash content remained nearly unchanged (~2 wt.%).

Elemental analysis (Table 1) confirmed that thermal treatment increased carbon from 53.81 wt.% (C-RAW) to 79.25 wt.% (C-SP) and ~77.85 wt.% (C-MWP). Hydrogen decreased from 6.50 wt.% (C-RAW) to 5.27 wt.% (C-SP) and 5.84 wt.% (C-MWP), while nitrogen decreased from 0.94 wt.% to 0.74 wt.% (C-SP) and 0.81 wt.% (C-MWP), consistent with dehydrogenation and partial volatilization of nitrogen-containing groups during carbonization. Sulfur was below the detection limit in all processed samples. Overall, cork-derived chars showed high carbonization efficiency, comparable for both SP and MWP pathways. XRF screening indicated only trace-level inorganic impurities in both biochars (see the Supplementary Materials, Table S7).

#### 2.1.2. Thermal Decomposition and Product Distribution

**Figure 1:**
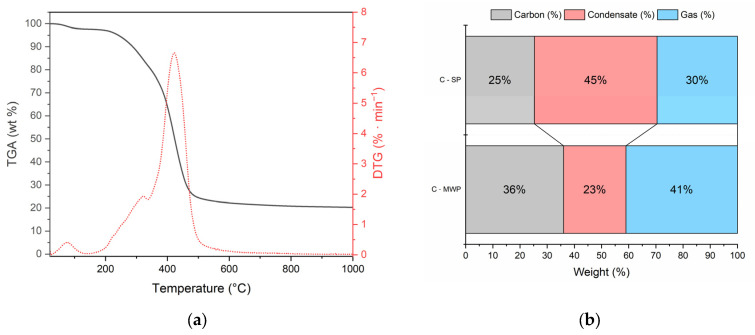
DTG curve of raw cork (**a**) and product distribution from different pyrolysis/activation routes (**b**). “Liquid” denotes the total condensate (aqueous pyrolysis water + organic fraction) collected gravimetrically; phases were not separated.

Thermogravimetric analysis (Figure 1a) of raw cork showed three decomposition steps: (i) minor weight loss below 120 °C due to moisture evaporation, (ii) main devolatilization between 250–450 °C with a DTG peak at ~380 °C, associated with hemicellulose/cellulose degradation and suberin decomposition, and (iii) gradual decomposition of lignin above 450 °C.

The distribution of pyrolysis products (Figure 1b) clearly demonstrated the effect of the heating mode. C-SP yielded 25% solid carbon, 45% condensate, and 30% gas. In contrast, C-MWP increased the carbon yield (36%) and gas fraction (41%) while reducing the condensate share to 23%. This confirms that MWP treatment promotes secondary cracking and gasification reactions, enhancing permanent gas formation at the expense of condensable products.

#### 2.1.3. Textural Properties

**Figure 2:**
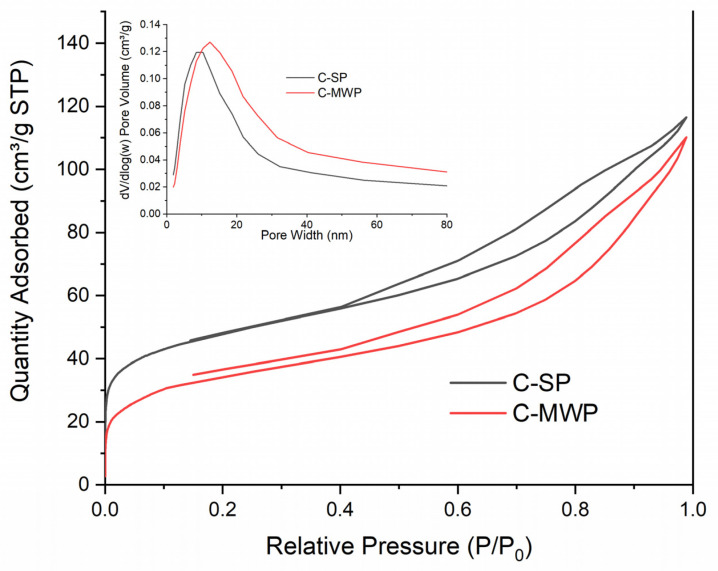
N_2_ adsorption–desorption isotherms (C-SP, C-MWP).

Textural parameters derived from N_2_ adsorption–desorption experiments are summarized in Table 2. SP char exhibited a higher specific surface area (174.5 m^2^·g^−1^) and total pore volume (0.18 cm^3^·g^−1^), whereas MWP char demonstrated reduced *S_BET_* (130.9 m^2^·g^−1^) and *V_net_* (0.17 cm^3^·g^−1^). Both chars were dominated by mesopores (0.14–0.15 cm^3^·g^−1^), while micropore volume was relatively low (0.02–0.04 cm^3^·g^−1^). The median micropore size shifted from 0.56 nm (C-SP) to 0.64 nm (C-MWP), suggesting partial widening of micropores during MWP treatment. The shift in median micropore size and the reduced micropore volume observed for the MWP sample (Figure 2) indicate a partial widening and collapse of the smallest pores, consistent with microwave-induced local overheating and restructuring of the carbon matrix.

The adsorption isotherms (Figure 2) correspond to type IV with H3 hysteresis loops, typical for mesoporous carbonaceous materials. The lower adsorption capacity of MWP char indicates a less developed porosity, consistent with the reduced BET surface area.

#### 2.1.4. FTIR Spectral Analysis

**Figure 3:**
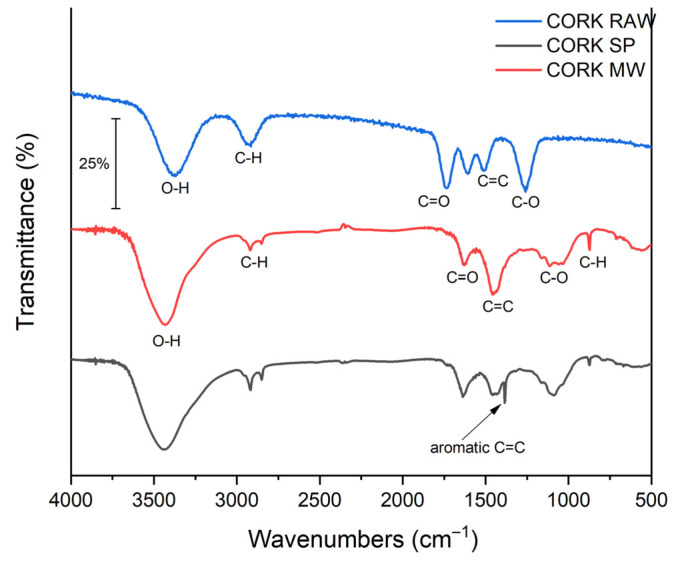
FTIR spectra of cork-derived biochars produced by SP and MWP.

FTIR spectra (Figure 3) validated the typical features of lignocellulosic-derived carbons. A broad O–H stretching band at 3200–3600 cm^−1^ is observed in both chars, with a higher transmittance (i.e., lower absorbance) for biochar produced with MW heating, indicating fewer hydroxyl-containing functionalities (phenolic, alcoholic and carboxylic groups). Bands at ≈2920 and ≈2850 cm^−1^ were assigned to aliphatic C–H stretching (–CH_2_– and –CH_3_) associated with lignin-derived structures [26,27]. A peak near ≈ 1700–1730 cm^−1^ corresponds to C=O stretching in carbonyl functionalities (esters, ketones and carboxylic acids) [28]. Features in the 1000–1300 cm^−1^ region were attributed to C–O stretching, while signals in the 1510–1580 cm^−1^ region were assigned to aromatic C=C skeletal vibrations.

Compared to SP, the C-MWP shows generally weaker absorbance in the O–H, C=O and C–O regions, indicating partial removal of oxygenated functional groups and a higher degree of aromatization/condensation of the carbon matrix. Notably, the band near ~1510–1580 cm^−1^ (aromatic C=C) [29] diminishes in MWP relative to SP, supporting a structural rearrangement toward a more condensed carbon matrix rather than an O–H/C–O assignment at ~1500 cm^−1^. These spectral changes are consistent with partial deoxygenation and increased condensation/aromatization of the carbon matrix during MWP [25].

For comparison, the FTIR spectrum of raw cork (Cork-raw) was also evaluated, showing characteristic O–H and C–O bands that diminish after pyrolysis, while the aromatic C=C region (1510–1580 cm^−1^) becomes more pronounced, confirming the structural transformation of cork during carbonization [30,31]. Compared with C-MWP, C-SP exhibits stronger O–H/C–O bands (~1000–1300 and ~3200–3600 cm^−1^), consistent with a higher density of oxygenated surface groups.

#### 2.1.5. Physico-Chemical Differences and Implications

In our case, SP-derived biochar showed higher surface area and porosity than its MWP counterpart, features commonly associated with improved adsorption performance. This is attributed to the gradual thermal decomposition and uniform carbonization, which promote the development of a stable and hierarchical pore structure [32].

In contrast, under the employed MW conditions, the C-MWP exhibited lower specific surface area and microporosity. This can be attributed to the rapid, localized heating inherent to MW treatment, which—without process optimization—may promote pore widening or local structural collapse and thereby reduce accessible porosity [33].

### 2.2. Effect of pH

The solution pH is one of the most influential factors affecting the adsorption of Cr(VI) onto different sorbents. It is well established that the adsorption efficiency of Cr(VI) decreases significantly with increasing pH, regardless of the nature of the adsorbent [34,35]. This trend is related both to the aqueous speciation of Cr(VI) and to the surface properties of the sorbent. At low pH (≈1–2), the dominant species in solution is HCrO_4_^−^, which is electrostatically attracted to the protonated sorbent surface. With increasing pH, CrO_4_^2−^ and Cr_2_O_7_^2−^ prevail, and due to electrostatic repulsion, their adsorption becomes less efficient [36,37].

Based on these considerations, the pH range 1.1–5.0 was selected for this study. Adsorption of Cr(VI) typically declines with pH; we focused on pH ≤ 5.0 because the largest removal differences and mechanistic insights for HCrO_4_^−^ vs. CrO_4_^2−^ occur in this range [6,38,39]. At pH 2.0, HCrO_4_^−^ is the dominant Cr(VI) species (with Cr_2_O_7_^2−^ at higher total chromate), and protonation of surface oxygen groups increases electrostatic attraction; redox to Cr(III) cannot be excluded.

Adsorption tests were carried out with 0.1 g biochar and 20 mL of 10 mg·L^−1^ Cr(VI) solution (solid-to-liquid ratio 5 g·L^−1^). The contact time was intentionally set to 45 min to prevent complete removal of Cr(VI) while allowing subsequent optimization for kinetic and equilibrium studies. Because Cr(VI) stability under strongly acidic conditions can be of concern, control experiments without biochar were performed under the same conditions (see Table S3). Monitoring of pH and Cr(VI) concentrations in these controls showed no significant changes over 45 min (e.g., at pH 2: *c*_0_ = 9.94 ± 0.9 mg·L^−1^; *c*_45_ = 10.08 ± 0.4 mg·L^−1^; change +1.41%), indicating that the observed loss of Cr(VI) in adsorption tests is attributable to interaction with the biochars.

**Figure 4:**
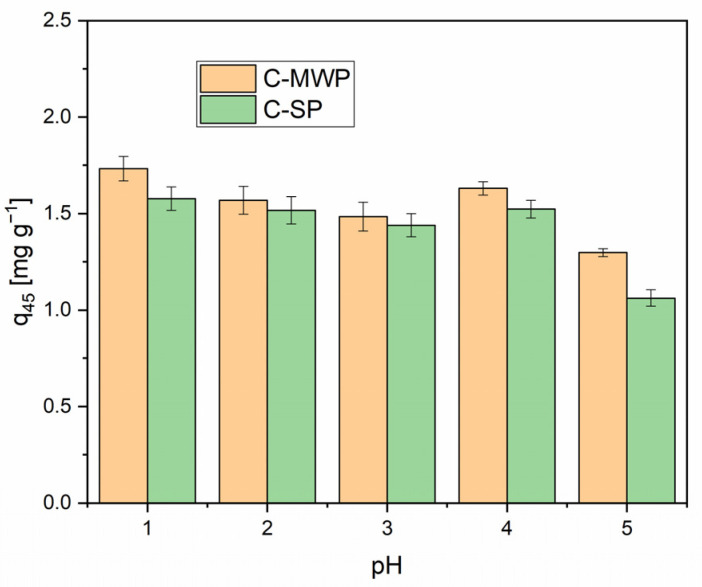
Effect of pH on Cr(VI) adsorption. Conditions: adsorbent dose = 0.1 g, *V* = 20 mL; *c*_0_ = 10 mg·L^−1^; pH = 1.1–5.0 (buffered solutions); contact time = 45 min; *T* = 22 ± 2 °C; rotation speed = 180 rpm. Data are presented as mean ± SD (*n* = 3). Error bars represent standard deviation.

As shown in Figure 4, the adsorption capacity was highest at pH 1.1 (C-SP: *q*_45_ = 1.44 ± 0.04 mg·g^−1^ corresponding to 72% removal; C-MWP *q*_45_ = 1.73 ± 0.06 mg·g^−1^ corresponding to 87% removal) and gradually decreased with increasing pH. A more pronounced drop was observed at pH 5.0, which is consistent with the general trend reported in both earlier and recent studies. Although the PFO model provided high R^2^ values, such empirical agreement does not uniquely identify the adsorption mechanism. PFO-type kinetics can result from a distribution of site energies or rate-limiting external mass transfer, and, therefore, mechanistic assignments require complementary data. Therefore, mechanistic claims are made cautiously and supported by complementary evidence (Elovich behavior, IPD and surface chemistry) [40,41].

### 2.3. Effect of Adsorbent Dose

To investigate the effect of adsorbent dose, Cr(VI) solutions at the optimum pH of 2.0 (stabilized with HCl/KCl solution) were used with an initial concentration of 40 mg·L^−1^, a solution volume of 20 mL, and a contact time of 45 min. Biochar masses of 0.02, 0.05, 0.1, 0.3, 0.5, and 0.7 g were tested.

**Figure 5:**
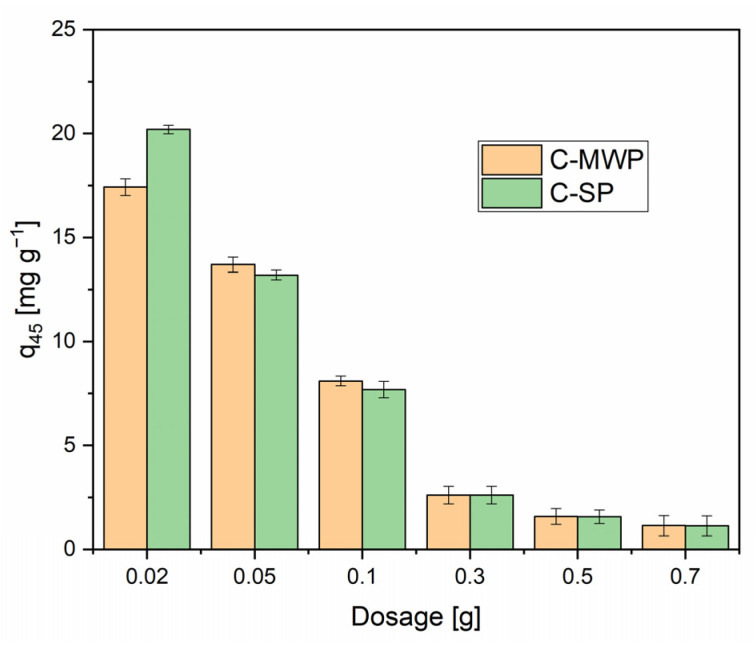
Effect of adsorbent dose on Cr(VI) adsorption. Conditions: *c*_0_ = 40 mg·L^−1^; *V* = 20 mL; adsorbent dose = 0.02–0.7 g; pH = 2.0 (buffered); contact time = 45 min; *T* = 22 ± 2 °C; rotation speed = 180 rpm. Data are presented as mean ± SD (*n* = 3). Error bars represent standard deviation.

As shown in Figure 5, increasing the adsorbent dosage improved the overall Cr(VI) removal efficiency. However, the specific adsorption capacity (*q*, mg·g^−1^) followed an inverse trend, with the highest values observed at the lowest dosage (0.02 g). This behavior is well documented and is generally attributed to the higher ratio of initial concentration to available active sites at lower doses, as well as to particle agglomeration at higher dosages, which can lead to site coverage and overlapping, thereby reducing the effective surface area [42,43].

Although the maximum adsorption capacity was achieved at 0.02 g of biochar, a dose of 0.05 g was selected for subsequent kinetic and equilibrium experiments. At too low a dose, adsorption may deviate from the linear region of the isotherm, which could result in non-representative data. In addition, the relative analytical error increases with decreasing removal of Cr(VI), particularly in colorimetric measurements, where small differences in absorbance can strongly affect accuracy. Therefore, 0.05 g was chosen as a compromise dose, ensuring sufficient sorption capacity as well as reproducible results [44,45,46].

### 2.4. Kinetic Study

The adsorption kinetics of Cr(VI) onto cork-based biochars were evaluated at pH 2.0 (*c*_0_ = 40 mg·L^−1^, *m* = 0.05 g, *V* = 20 mL) using four common models: pseudo-first-order (PFO), pseudo-second-order (PSO), Elovich, and intraparticle diffusion (IPD) [40,47]. Full numerical fits, parameter standard errors and residuals are provided in Supplementary Materials (Figures S3 and S4; Table S4). Kinetic experiments show a rapid initial uptake followed by a plateau. The change in uptake between 60 and 90 min was small (maximum observed change ≤ 4.5% across tested conditions; see Supplementary Materials, Table S5), supporting the use of 60 min as the equilibrium contact time for the isotherm determinations.

**Figure 6:**
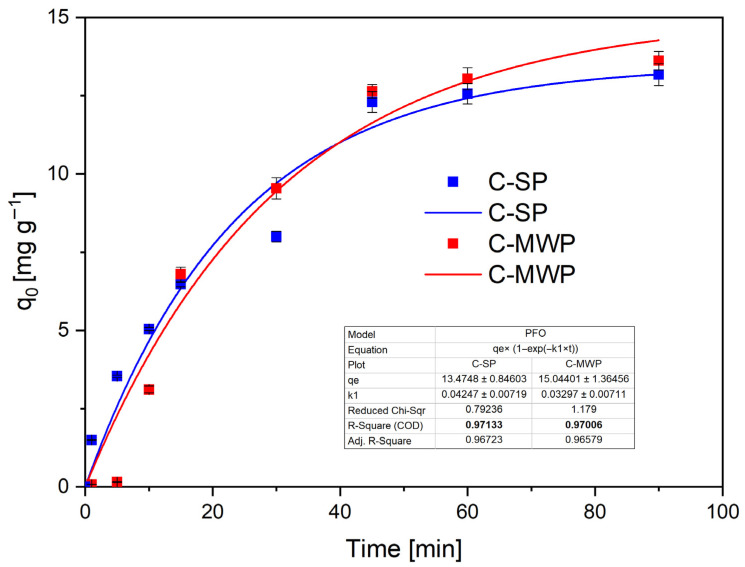
PFO model fitted to Cr(VI) adsorption data (C-SP, C-MWP). Conditions: *c*_0_ = 40 mg·L^−1^; *V* = 20 mL; *m* = 0.05 g; pH = 2.0 (buffered); contact time = 0–90 min; *T* = 22 ± 2 °C; rotation speed = 180 rpm. Data are presented as mean ± SD (*n* = 3). Error bars represent standard deviation. Fit method: nonlinear regression (OriginPro 2023).

The PFO model (Figure 6) provided a good fit for both materials (C-SP: R^2^ ≈ 0.97; C-MWP: R^2^ ≈ 0.97), with estimated equilibrium capacities *qₑ* of 13.5 mg·g^−1^ (C-SP) and 15.0 mg·g^−1^ (C-MWP) and estimated rate constants k_1_ in the range 0.03–0.05 min^−1^.

**Figure 7:**
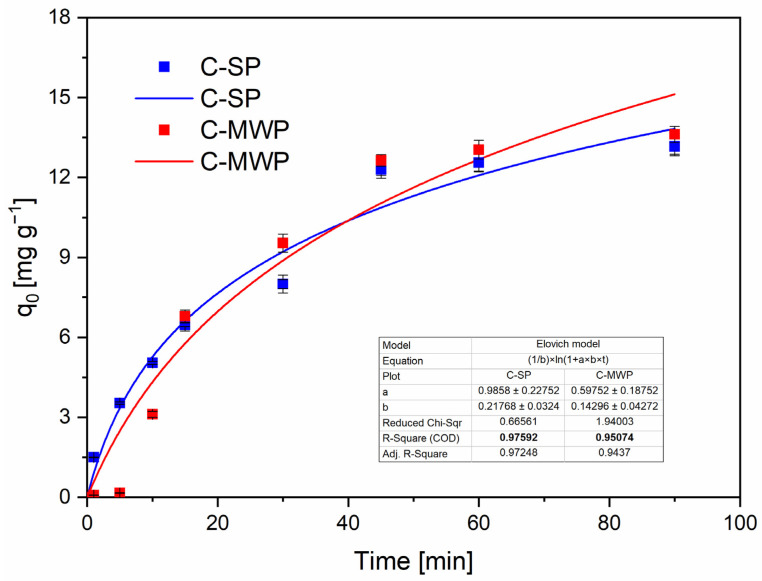
Elovich model fitted to Cr(VI) adsorption data (C-SP, C-MWP). Conditions: *c*_0_ = 40 mg·L^−1^; *V* = 20 mL; *m* = 0.05 g; pH = 2.0 (buffered); contact time = 0–90 min; *T* = 22 ± 2 °C; rotation speed = 180 rpm. Data are presented as mean ± SD (*n* = 3). Error bars represent standard deviation. Fit method: nonlinear regression (OriginPro 2023).

The Elovich model (Figure 7) returned high R^2^ values (C-SP: ≈0.976; C-MWP: ≈0.951), consistent with adsorption on a surface with an energetic distribution of active sites and with a rate that decreases with progressive surface coverage. The Elovich initial adsorption parameter (α) was higher for C-SP, which matches the faster early uptake observed experimentally [48].

In the Supplementary Materials, Table S6 presents a quantitative comparison of the experimentally observed uptake qₑ_,exp_ (*t* = 60 min; mean ± SD, *n* = 3) and the model-predicted *q_PFO_*(60) and *q_Elovich_*(60). Both kinetic models show high R^2^ and the absolute percent deviations between model predictions and measured values are small (|%dev| ≤ 15%), indicating good quantitative agreement. Although both models provide strong empirical descriptions (high R^2^ and low deviations), kinetic fits alone cannot establish the underlying sorption mechanism (e.g., physisorption vs. chemisorption). A definitive assignment requires corroboration by surface/speciation analyses such as XPS, pHpzc determination, activation-energy analysis, or post-contact chromium speciation (Cr(III) quantification), which we recommend for future work [40,47].

**Figure 8:**
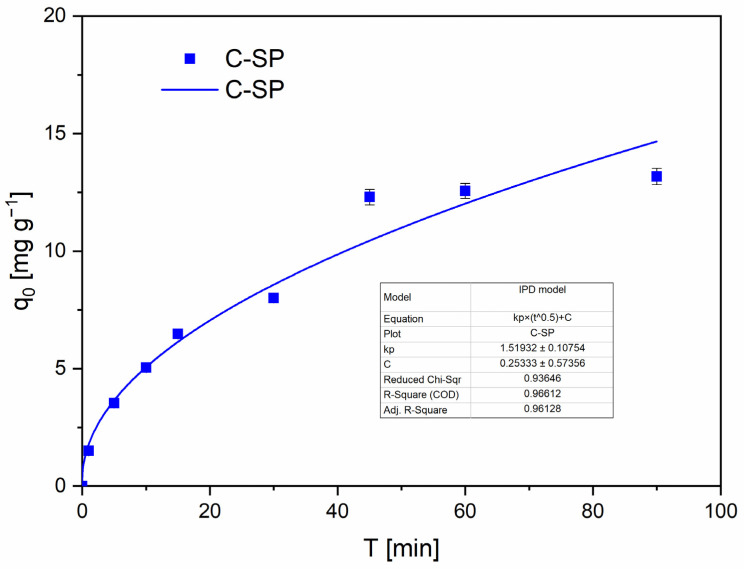
IPD plots (C-SP). Conditions: *c*_0_ = 40 mg·L^−1^; *V* = 20 mL; *m* = 0.05 g; pH = 2.0 (buffered); contact time = 0–90 min; *T* = 22 ± 2 °C; rotation speed = 180 rpm. Data are presented as mean ± SD (*n* = 3). Error bars represent standard deviation. Fit method: nonlinear regression (OriginPro 2023).

IPD analysis confirms that pore diffusion contributes to the overall uptake. For C-SP the IPD plot (Figure 8) shows a strong linear region with a positive intercept (C ≠ 0, see Table S4), which is commonly interpreted as evidence of combined boundary-layer (film) and pore diffusion contributions; for C-MWP the IPD fit is weaker and appears multi-stage, consistent with sequential transport regimes [49,50].

Taken together, the kinetic evidence points to rapid surface-accessible uptake together with contributions from external mass transfer and intraparticle diffusion and confirms energetic heterogeneity of adsorption sites; however, we avoid attributing the process solely to physisorption or chemisorption based on kinetic fits alone. Similar multi-model approaches have been used in the literature for Cr(VI)/biochar systems and are consistent with combined adsorption/redox behaviors reported elsewhere [6].

### 2.5. Adsorption Isotherms

The equilibrium adsorption data were evaluated using several common isotherm models: Freundlich, Langmuir, Dubinin–Radushkevich (D–R) and Redlich–Peterson (R–P). Full numerical fits, parameter standard errors and residuals are provided in Supplementary Materials (Figures S5–S8; Table S4).

The Freundlich model provided a good fit for C-SP, indicating a heterogeneous surface and multilayer adsorption behavior typical of porous, chemically heterogeneous biochars [51]. Although the R–P model produced a high R^2^ for C-SP, its fit parameters exhibited very large relative uncertainties, indicating poor parameter identifiability and limiting mechanistic interpretation [52,53]. For C-MWP, none of the tested models produced an acceptable and physically meaningful fit. Therefore, isotherm-based mechanistic conclusions are not drawn for C-MWP. The Langmuir and D-R models also failed to describe the data for both materials reliably and are not used to report single-point capacities in the main text.

The experimentally observed maximum uptake values (*qmax*,*obs*), defined as the highest measured equilibrium adsorption capacity (*qₑ*) within the concentration range *c*_0_ = 5–150 mg·L^−1^, were 32.75 mg·g^−1^ for C-SP and 35.91 mg·g^−1^ for C-MWP. Experimental conditions were as follows: pH 2.0, adsorbent dosage 2.5 g·L^−1^, and equilibrium time *t_e_* = 60 min. Model fitting parameters for Langmuir, Freundlich, R–P, and D–R isotherms are summarized in the Supplementary Materials Table S4. Since the Langmuir model yielded low R^2^ values and non-physical parameters, *qmax*,*obs* is reported as a conservative comparative metric.

**Figure 9:**
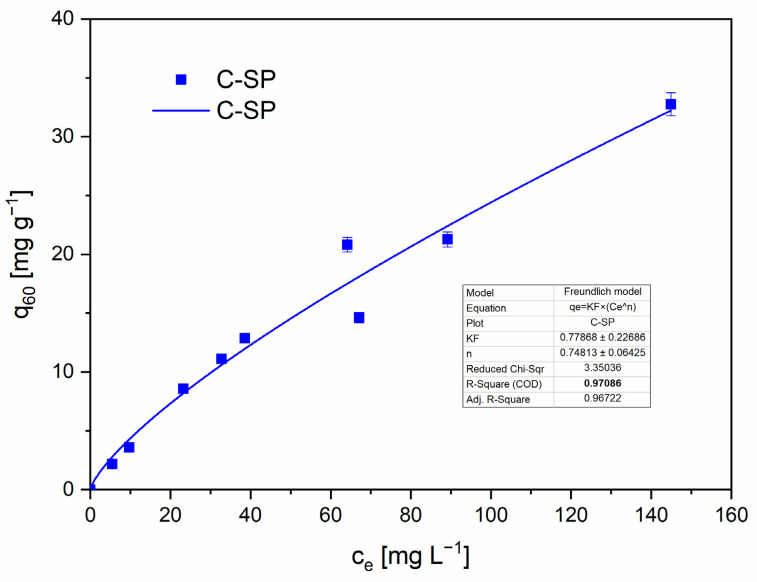
Freundlich adsorption isotherm for Cr(VI) removal C-SP. Conditions: *cₑ* = 5–150 mg·L^−1^; *V* = 20 mL; *m* = 0.05 g; pH = 2.0 (buffered); contact time = 60 min; *T* = 22 ± 2 °C; rotation speed = 180 rpm. Data are presented as mean ± SD (*n* = 3). Error bars represent standard deviation. Fit method: nonlinear regression (OriginPro 2023).

The Freundlich fit for C-SP is presented in Figure 9 with the corresponding parameters.

### 2.6. Linking Biochar Properties to Adsorption Behavior

The differences in Cr(VI) uptake between C-SP and C-MWP can be rationalized by their distinct textural and surface-chemical properties. C-SP displays a larger specific surface area (*S_BET_* = 174.5 m^2^·g^−1^), and FTIR spectra indicate relatively stronger bands attributable to oxygenated, protonatable groups (e.g., –OH) compared with C-MWP (*S_BET_* = 130.9 m^2^·g^−1^). Under strongly acidic conditions, these functionalities are readily protonated and thus promote electrostatic attraction of oxyanionic Cr species (e.g., HCrO_4_^−^), and can also participate in specific complexation or surface-mediated redox pathways [5,6,54].

However, specific surface area alone does not fully determine anion uptake by heterogeneous biochars. To provide a more mechanistic perspective, we estimated apparent active-site densities by converting the experimentally observed maximum uptakes (*qmax*,*obs*) into site counts and normalizing by *S_BET_* (calculation details and formula are given in the Supplementary Materials). The resulting apparent site densities (see the Supplementary Materials, Table S2) are 2.2 ± 0.07 sites·nm^−2^ for C-SP (*qmax*,*obs* = 32.7 ± 0.98 mg·g^−1^) and 3.2 ± 0.10 sites·nm^−2^ for C-MWP (*qmax*,*obs* = 35.9 ± 1.07 mg·g^−1^). For reference, the observed *qmax*,*obs* values (32.7–35.9 mg·g^−1^) fall within the range reported for activated carbons and other lignocellulosic biochars tested under comparable acidic conditions (see the Supplementary Materials, Table S1). Thus, despite its lower *S_BET_*, C-MWP exhibits a higher density of adsorption sites per unit area, while C-SP provides a larger total accessible area.

This apparent dichotomy suggests that the observed uptake differences reflect a trade-off between total accessible surface (favoring C-SP) and local site density/surface functionalization per unit area (favoring C-MWP). Practically, C-SP’s greater total area can increase the absolute number of accessible sites and favor pore-filling contributions (consistent with the Freundlich-type behavior observed for C-SP), whereas the denser surface functionalization of C-MWP can lead to higher per-area uptake but may reduce pore accessibility or alter energetic heterogeneity, explaining the poorer physical interpretability of some fitted isotherm models for C-MWP. These modality-dependent effects are consistent with prior comparative studies of SP vs. MWP and with mechanistic reports on Cr(VI) uptake on carbonaceous adsorbents [7,55].

We emphasize important limitations of the site-density analysis: the calculated values are apparent and assume a 1:1 stoichiometry between adsorbed Cr atoms and surface sites. They are normalized by *S_BET_* measured by N_2_ physisorption and therefore assume that the BET area is accessible to the adsorbate. If Cr(VI) is partially reduced to Cr(III) with subsequent precipitation, or if multisite complexation occurs, the apparent site densities will not represent true chemical site counts. For definitive mechanistic assignment (electrostatic adsorption vs. specific complexation vs. surface-mediated redox and precipitation), complementary analyses such as XPS of the adsorbent surface after uptake, pHpzc determination, potentiometric/Boehm titration of surface groups, and total-Cr vs. Cr(VI) mass balances in solution would be required.

In summary, our combined textural, spectroscopic, and site-density data show that pyrolysis modality (SP vs. MWP) substantially alters both the amount and the per-area distribution of adsorption sites on cork biochars, producing measurable differences in Cr(VI) uptake that cannot be explained by *S_BET_* alone (see Supplementary Materials, Table S2). At pH 2.0, HCrO_4_^−^ predominates and surface protonation enhances electrostatic attraction, consistent with the observed higher uptake at low pH. At the same time, surface-mediated reduction of Cr(VI) to Cr(III) cannot be excluded; definitive discrimination between electrostatic binding and redox requires XPS/Cr speciation and pHpzc measurements, which were beyond the scope of this study. Elemental analysis indicates lower oxygen functionality for MWP relative to SP (consistent with FTIR), which is qualitatively in line with the observed differences. In line with this distinction, several studies have directly observed partial reduction of Cr(VI) to Cr(III) on carbonaceous surfaces under acidic conditions using XPS/FTIR: on biochar and activated carbons, the removal proceeds via adsorption-coupled reduction, with surface oxygenated/phenolic groups acting as reductants, and Cr(III) detected on the spent sorbent surface [7,8,9]. Reports further indicate that the balance between electrostatic attraction (HCrO_4_^−^ at low pH) and surface-mediated reduction depends on surface functionality and matrix; modified carbons and biochars with richer redox-active moieties show stronger Cr(VI)→Cr(III) signatures by XPS [25]. This qualitative trend is consistent with the FTIR evidence of stronger O–H/C–O signatures for C-SP.

When benchmarked against literature data for Cr(VI) adsorption on lignocellulosic biochars (Table S1), the experimentally observed maximum capacities of the cork-derived materials (32.7–35.9 mg·g^−1^) fall within the range commonly reported for unmodified biochars tested under comparable acidic conditions (typically 20–120 mg·g^−1^) [4,56,57,58,59,60]. These values confirm that both C-SP and C-MWP exhibit adsorption efficiencies consistent with previously studied lignocellulosic carbons, despite differences in feedstock type and pyrolysis conditions. Compared with chemically modified or metal-loaded biochars, which can reach capacities exceeding 100 mg·g^−1^ [59,60] the present unmodified cork-based materials demonstrate competitive performance, highlighting the importance of feedstock selection and inherent surface chemistry.

## 3. Materials and Methods

### 3.1. Biomass Preparation

Cork biomass (denoted as C) used for the preparation of biochar was obtained from industrial cork wood residues provided by the Research Institute of Environmental Technologies, VŠB—Technical University Ostrava.

The biomass was mechanically milled using a knife mill TESTCHEM LMN-180 (Testchem, Radlin, Poland) equipped with a 5.0 mm sieve. It was then dried at 105 °C until constant weight in a UF 260 oven (Memmert, Schwabach, Germany). Sieving was performed using a Retsch AS200 oscillating shaker (Retsch GmbH, Düsseldorf, Germany) with Retsch sieves, and the particle size fraction of 3.0–4.0 mm was selected for pyrolysis experiments.

### 3.2. Pyrolysis Processes

MWP and SP were conducted at the Institute of Environmental Technologies, VŠB—Technical University of Ostrava, following experimental protocols based on previous studies by Jankovská et al. (2024), Vaštyl et al. (2022), and Grycová et al. (2018) [25,61,62]. The specific operating conditions are described below.

MWP was carried out in a closed system equipped with a quartz reactor (500 mL) containing 50 g of biomass and 5 g of previously prepared biochar (denoted as C-P). An auxiliary starter biochar (5.0 g, produced by SP at 600 °C, heating rate 10 °C·min^−1^, 1 h, N_2_ atmosphere) was added to the MW runs as a MW absorber to facilitate initial coupling and reduce sparking. The starter corresponded to approximately 10 wt.% of the total feed mass and remained mixed with the feed during pyrolysis. Its use ensured more uniform energy distribution and safer ignition under MW irradiation, an approach consistent with earlier reports on microwave-assisted biomass conversion [25].

Prior to MW heating, the system (see Supplementary Materials, Figure S1) was purged with N_2_ for 5 min (3 L·min^−1^) to establish an inert atmosphere. MWP was conducted at a power of 400 W for 30 min. Due to the nature of the equipment, MW conditions are reported in terms of applied power rather than controlled reactor temperature. After completion, the biochar was cooled under an N_2_ stream to room temperature.

SP was designed to be comparable to MWP, maintaining identical parameters: biomass weight (50 g), reactor volume (500 mL), N_2_ flow rate (3 L·min^−1^), and cooling/condensation system. The temperature ramp was set to 10 °C·min^−1^, with a final temperature of 600 °C and a residence time of 1 h. The process was carried out under an inert atmosphere, and the resulting biochar was cooled under N_2_ to room temperature. The experimental setup is illustrated in the Supplementary Materials, Figure S2.

While SP was temperature-programmed (final temperature = 600 °C), MWP was operated under power control (400 W), resulting in different apparent thermal regimes. Full operational parameters are provided in the Supplementary Materials. We note that reliable in situ temperature profiling in the MW field was not available in the present setup. Future work will implement IR thermography to correlate power input with surface temperatures.

### 3.3. Post-Pyrolysis Biochar Treatment

Each sample was first washed with 1 L of boiling deionized water, followed by several rinses with cold deionized water until a neutral pH was reached (typically after five washing cycles). Filtration was performed using a Millipore WP6122050 Chemical Duty Vacuum Pump (MilliporeSigma, Darmstadt, Germany) and a Büchner funnel with Whatman No. 3 filter paper (Cytiva, Marlborough, MA, USA). The washed material was dried at 105 °C until constant weight and stored in a desiccator.

The final product was sieved through standard laboratory sieves, and the particle size fraction < 0.1 mm was used for further experiments.

### 3.4. Biochar Characterization

All analyses were carried out at the Research Institute of Environmental Technologies, VŠB—Technical University of Ostrava. Biochar characterization included proximate and elemental analyses, thermogravimetric analysis (TGA), nitrogen adsorption (BET) surface area and porosity measurements, and Fourier transform infrared spectroscopy (FTIR).

Proximate analysis (moisture, volatile matter, ash, fixed carbon) was performed using a LECO TGA 701 (LECO Corporation, St. Joseph, MI, USA) instrument according to ASTM D7582 [63]. Moisture content was determined as the weight loss at 105 °C until constant weight. Proximate values for raw cork biomass are reported as received (i.e., including natural moisture), whereas biochar values are expressed on a dry weight basis.

Elemental analysis (CHNS) was conducted on a LECO CNS628 analyzer (LECO Corporation, St. Joseph, MI, USA) (values reported on a dry weight basis). Thermogravimetric analysis (TGA) was applied to monitor weight changes in raw samples as a function of temperature and to assess thermal stability relevant to pyrolysis processes. Elemental O was not directly measured; estimation by difference is known to be unreliable for biochars due to mineral oxygen and bound water and was therefore not used to compute O/C. Bulk inorganic impurities were screened by XRF (see the Supplementary Materials, Table S7); all monitored heavy metals were below detection or present at trace levels. Activation-energy calculations (e.g., KAS/FWO) were not attempted; TGA/DTG was used qualitatively to characterize devolatilization and char formation.

Surface area and pore structure were determined by nitrogen adsorption (BET method). Functional groups were identified using FTIR. Representative instrument parameters and raw data are provided in the Supplementary Materials (Biochar characterization).

### 3.5. Batch Adsorption Experiments

A series of batch adsorption experiments was carried out to evaluate the adsorption performance of the biochars. The results were analyzed using mathematical models describing adsorption kinetics and equilibrium. The experimental plan was designed to assess the effect of pH and adsorbent dose, to investigate adsorption kinetics at the optimum pH, and to determine equilibrium parameters.

First, the effect of pH on adsorption was studied to identify the optimum value for further experiments. To maintain constant pH, different buffer systems were applied: HCl/KCl solution for pH 1.1–2.0 and citrate/Na_2_HPO_4_·2H_2_O for pH 3.0–5.0. KCl was used as a background electrolyte to maintain a stable ionic strength (0.1 M) during pH adjustment, improving reproducibility across screening and isotherm/kinetic tests. The buffers were employed to enable rapid, reproducible preparation of solutions and to ensure pH stability during prolonged contact with the sorbents. The pH was monitored using a Hanna EDGE pH meter (Hanna Instruments, Prague, Czech Republic). Adsorption tests were performed with 0.1 g of biochar and 20 mL of Cr(VI) solution in 100 mL polyethylene centrifuge tubes (corresponding to 5 g·L^−1^ biochar). The initial Cr(VI) concentration was 10 mg·L^−1^, and the contact time was 45 min. To verify the stability of Cr(VI) under strongly acidic conditions, control experiments without biochar were also carried out. Blank tests without biochar showed no measurable loss of Cr(VI) over 45 min under the same acidic conditions (see the Supplementary Materials, Table S3), confirming solution stability during the screening step. The pH and Cr(VI) concentration were monitored both in the initial solutions and after 45 min, and no changes were observed. Based on these results, pH 2.0 was selected as optimum for subsequent experiments.

The effect of biochar dose was then studied at pH 2.0 using the following masses: 0.02, 0.05, 0.1, 0.3, 0.5, and 0.7 g. The initial Cr(VI) concentration was 40 mg·L^−1^, the solution volume 20 mL, and the contact time 45 min. From these results, the optimum adsorbent dose was determined for kinetic and equilibrium experiments.

Adsorption kinetics experiments were investigated at contact times of 0, 1, 5, 10, 15, 30, 45, 60, and 90 min. The biochar mass was 0.05 g with 20 mL of adsorbate in 100 mL polyethylene centrifuge tubes (2.5 g·L^−1^ biochar). The initial Cr(VI) concentration was 40 mg·L^−1^ at pH 2.0.

Equilibrium adsorption experiments were conducted at initial Cr(VI) concentrations of 5, 10, 20, 30, 40, 60, 80, 100, and 150 mg·L^−1^. Equilibrium contact time for isotherm experiments was set to 60 min. This choice was based on kinetic tests (contact times: 0, 1, 5, 10, 15, 30, 45, 60 and 90 min), which showed that *qₜ* values reached a plateau by 60 min under the tested conditions. Representative kinetics and numerical comparisons between *q* at 60 and 90 min are provided in the Supplementary Materials (Figure S9; Table S5). In all cases, the pH was adjusted to 2.0 using the HCl/KCl solution. The buffer was employed to enable rapid, reproducible preparation of solutions and to ensure pH stability during prolonged contact with the sorbents. All reagents used were of analytical grade.

Control experiments without biochar were performed to verify the reliability of adsorption data and exclude potential contamination of Cr(VI) from the experimental setup or materials (see the Supplementary Materials Table S3). Speciation analysis Cr(VI)/Cr(III), pHpzc determination and surface-sensitive techniques (XPS) were not performed in this study and are recommended for follow-up work to confirm mechanistic pathways. The pH was measured before and after adsorption to monitor its stability during the experiments.

#### Kinetic and Equilibrium Models

To better understand the adsorption mechanisms of Cr(VI) onto biochar, experimental data were analyzed using selected mathematical models describing adsorption kinetics and equilibrium.

Kinetic data, which provide insights into the adsorption rate and controlling mechanisms, were evaluated using four models: PFO, PSO, Elovich equation, and IPD models. can indicate rate-controlling steps and provide indirect clues about surface heterogeneity; however, they do not by themselves distinguish physisorption from chemisorption. For quantitative assessment of kinetic fits, the experimentally measured adsorption capacity *qₑ*_,*exp*_ (arithmetic mean of *n* = 3 replicates) at *tₑ* = 60 min was compared with model-predicted values at the same time (*q_PFO_*(60) and *q_Elovich_*(60)). Fitted model parameters (including *qₑ*_,*fit*_, k_1_, α, β are provided in Table S4. Time-matched model predictions and percentage deviations are reported in Table S6.

Equilibrium data were fit using four isotherm models: the Langmuir isotherm (monolayer adsorption on a homogeneous surface), the Freundlich isotherm (multilayer adsorption on a heterogeneous surface), the R–P isotherm (a hybrid combining features of Langmuir and Freundlich models), and the D–R isotherm (characterizing adsorption on microporous materials with a uniform distribution of activation energies).

Mathematical formulations of all kinetic and isotherm models, including parameter definitions and constants, are provided in the Supplementary Materials (Kinetic and equilibrium models).

### 3.6. Methodology for the Determination of Cr(VI)

All working solutions with different Cr(VI) concentrations were prepared by successive dilution of a 1 g·L^−1^ stock solution (K_2_Cr_2_O_7_ p.a. Penta, Prague, Czech Republic) in double-distilled water. Fresh solutions were prepared for each experiment, and the initial Cr(VI) concentration of each working solution was verified spectrophotometrically prior to use.

The initial Cr(VI) concentrations in model solutions and the equilibrium residual concentrations after biosorption were determined spectrophotometrically using 1,5-diphenylcarbazide (DPC) as the complexing reagent and measured with a DR 2800 spectrophotometer (Hach Lange GmbH, Düsseldorf, Germany). The absorbance of the resulting purple complex was read at 545 nm. Blank controls (biochar + buffer, no Cr(VI)) were prepared and processed identically to adsorption samples and analyzed by the DPC method. No measurable absorbance at 545 nm was observed for these blanks (mean absorbance at or below instrumental noise; *n* = 3). For high-concentration samples (initial *c*_0_ ≥ 40 mg·L^−1^) filtrates were diluted 100-fold prior to DPC analysis. Blank filtrates (biochar + buffer, no Cr) were measured at the same dilution (*n* = 3) and showed negligible absorbance at 545 nm, indicating that biochar leachates do not interfere with the DPC-based Cr(VI) determination at the applied 100× dilution. Control experiments monitoring Cr(VI) stability in the chosen buffer (no adsorbent) showed no significant change in absorbance over the experimental timeframe (see the Supplementary Materials Table S3).

All adsorption experiments were performed in triplicate, and results are reported as arithmetic mean ± standard deviation (SD, *n* = 3). The analytical precision of the DPC spectrophotometric method in our laboratory was RSD ≈ 3%.

## 4. Conclusions

This work provides a focused, like-for-like comparison of cork-derived biochars (C-SP and C-MWP) prepared from the same feedstock. Textural and chemical differences were confirmed (C-SP: higher *S_BET_* and stronger O–H/C–O signatures; C-MWP: partial aromatization and less-developed texture), yet performance trends were not dictated by surface area alone. Under acidic conditions, C-MWP reached a slightly higher observed capacity than C-SP (*qmax*,*obs* = 35.9 vs. 32.7 mg·g^−1^ at pH 2.0), and at pH 1.1 the 45-min removals were 87% (1.73 mg·g^−1^) for C-MWP and 72% (1.44 mg·g^−1^) for C-SP. Kinetics were fast, with PFO R^2^ ≈ 0.97 for both materials and Elovich R^2^ ≈ 0.976/0.951; the ≤4.5% difference between 60 and 90 min supports the choice of tₑ = 60 min. For equilibrium, Freundlich model provided the best fit to the data for C-SP, whereas classical models did not yield physically meaningful parameters for C-MWP; we therefore report experimental capacities (*qmax*,*obs*) rather than relying on extrapolated model values. Together, the data indicate that pore architecture and surface chemistry co-govern Cr(VI) uptake beyond total surface area.

The main advantages of this work include the direct head-to-head comparison of SP and MWP biochars from an identical cork feedstock, rapid uptake enabling short contact times, and very low inorganic impurities, consistent with cork’s inherently low ash content. A concise comparison with literature data (SBET, capacity, and test conditions) is provided for context. Despite these strengths, several limitations should be acknowledged: no surface or speciation analyses (XPS, pHpzc, or Cr(VI)/total-Cr balances) were performed to confirm mechanistic assignments; regeneration cycles and real-wastewater validation were not included; potential leaching and long-term stability were not evaluated; microwave process parameters (temperature mapping) were not resolved; and no activation-route comparison (CO_2_/steam/KOH) or cost assessment was undertaken.

Future work will therefore focus on applying XPS and Cr speciation analyses to clarify the contribution of electrostatic adsorption versus surface-mediated redox processes, as well as on regeneration, long-term stability, and real-matrix testing to evaluate the practical potential of cork-based biochars for sustainable Cr(VI) remediation.

## Figures and Tables

**Table 1:** Proximate and elemental composition.

Material	Moisture (wt.%)	Ash (wt.%)	Volatile Matter (wt.%)	Fixed Carbon (wt.%)	C (wt.%)	H (wt.%)	N (wt.%)	S (wt.%)
C-RAW	3.5	2.58	78.85	15.07	53.81	6.50	0.94	<0.0002
C-SP	2.80	1.98	12.08	83.14	79.25	5.27	0.74	n. a.
C-MWP	2.45	2.45	11.07	84.03	77.85	5.84	0.81	n. a.

Note: n. a.—not analyzed.

**Table 2:** Textural parameters of cork-derived biochars (Data are presented as mean ± SD (*n* = 2)).

Material	*S_BET_* (m^2^·g^−1^)	*V_net_* (cm^3^·g^−1^)	*V_mic_* (cm^3^·g^−1^)	*V_meso_* (cm^3^·g^−1^)	Median Micropore Size (nm)
C-SP	174.5 ± 0.11	0.18 ± 0.017	0.04 ± 0.0024	0.14 ± 0.027	0.559
C-MWP	130.9 ± 0.27	0.17 ± 0.019	0.02 ± 0.0014	0.15 ± 0.016	0.635

## Data Availability

The original contributions presented in this study are included in the Supplementary Materials. Further inquiries can be directed to the corresponding author.

## Supplementary Materials

The following supporting information can be downloaded at: https://www.mdpi.com/article/10.3390/molecules30234501/s1, Refs. [63,64,65,66,67,68,69,70,71,72,73,74] are cited in the Supplementary Materials.
