# Supplementary material for: Removal of Hexavalent Chromium from Water Using Cork-Based Biochars Obtained via Slow and Microwave Pyrolysis
Source: Molecules. 2025 Nov 21;30(23):4501. doi: 10.3390/molecules30234501 (PMC12693453; doi:10.3390/molecules30234501)
Supplement: Supplementary file 1 [file molecules-30-04501-s001.zip › molecules-3930011-supplementary.pdf]

Supplementary Materials

Biochar characterization

Proximate analysis. Proximate analysis of the cork samples was performed following ASTM D7582 [63] using a LECO TGA 701 thermogravimetric analyzer (LECO Corporation, Michigan, USA). This protocol quantifies moisture content, volatile matter, fixed carbon, and ash, all reported on a mass basis (wt.%). Measurements were carried out using a controlled temperature program to sequentially separate the constituent fractions: moisture was determined by low temperature drying, volatile matter was quantified during the programmed temperature increase, fixed carbon was calculated by difference from the remaining solid fraction, and ash content was obtained by oxidative combustion at elevated temperature. This analysis provides a comprehensive characterization of sample composition and thermal behavior.

Elemental analysis (CHNS). Carbon, hydrogen, nitrogen and sulfur contents were determined by high-temperature combustion (>1,000 °C) in an O_2 atmosphere. Organic constituents were fully oxidized to CO_2, H_2O, SO_2 and NOx; nitrogen oxides were subsequently reduced in a reduction furnace, and the resulting gases were detected by a thermal conductivity detector (TCD). Helium (He) was used as the carrier gas. Analyses were performed on a LECO CNS628 analyzer (LECO Corporation, Michigan, USA).

Micro thermogravimetric analysis (Micro-TGA). Micro TGA was employed to assess mass changes of the raw (RAW) material as a function of temperature, thereby evaluating its thermal stability and suitability for pyrolysis. Measurements were carried out on a STA 300 instrument (Hitachi, Japan). Samples were placed in α-Al_2O_3 crucibles and exposed to a nitrogen flow of 100 $cm^3{\cdot}min^{-1}$. Heating was performed at a rate of 10 $°C{\cdot}min^{-1}$ up to 800 °C.

N_2 physisorption (BET). Specific surface area was determined by the Brunauer–Emmett–Teller (BET) method using N_2 physisorption at cryogenic temperature. Prior to analysis, samples were degassed under vacuum (<1 Pa) at 350 °C for 48 h. Adsorption measurements were recorded at −196 °C on a 3Flex surface area analyzer (Micromeritics, USA). Surface area calculations were based on adsorption isotherms measured up to a relative pressure p/p_0 = 0.99. Isotherms were interpreted according to the IUPAC classification scheme for adsorption types. [64,65]

Fourier-transform infrared spectroscopy (FTIR). FTIR spectroscopy was employed to identify surface functional groups in the biochars. Spectra were recorded with a Nicolet iS50 FT-IR spectrometer (Thermo Fisher Scientific, Wisconsin, USA) using the KBr pellet technique. Each spectrum is the average of 32 scans with background subtraction and software correction performed using OMNIC software (Thermo Fisher Scientific, Wisconsin, USA).

Kinetic and Equilibrium Models

The pseudo-first-order kinetic model (PFO) assumes that the rate of adsorption is proportional to the difference between the equilibrium adsorption capacity and the amount of adsorbate at a given time [66]. This model is expressed by the equation S1 [67]

$$q_t = q_e(1 - e^{-k_1 t}) \qquad \text{(S1)}$$

where:

q_t – amount of adsorbate at time t ($m^2{\cdot}g^{-1}$),

q_e – equilibrium adsorption capacity ($mg{\cdot}g^{-1}$),

k_1 – PFO rate constant (min^{-1}).

The pseudo-second-order model (PSO) assumes that the rate-limiting step of the adsorption process can be chemisorption. This involves interactions between the adsorbate and adsorbent based on electron sharing or exchange, leading to stronger chemical bonding [68]. Compared to the PFO model, it often provides a more accurate description of the adsorption mechanism [69].

PSO is described by Equation S2 [67]:

$$q_t = \frac{(q_e{}^2 k_2 t)}{(1+k_2 q_e t)} \qquad \text{(S2)}$$

where:

k_2 – PSO rate constant (g·mg^{-1}·min^{-1}).

All other variables are as previously defined.

The Elovich kinetic model is based on the assumption that during the adsorption process the activation energy increases gradually and the adsorbent surface is considered homogeneous [70] It is expressed by the equation S3 [67]:

$$q_t = \frac{1}{\beta} \ln{(1 + \alpha\beta t)} \qquad \text{(S3)}$$

where:

α – initial adsorption rate (m^2·g^{-1}·min^{-1}),

β – desorption constant (m^2·g^{-1}).

All other variables are as previously defined.

The intra-particle diffusion (IPD) model (Weber–Morris) was used to evaluate the contribution of intra-particle diffusion to the overall adsorption process. The model is expressed as equation S4:

$$q_t = k_{id} t^{1/2} C \qquad \text{(S4)}$$

where:

q_t – amount of Cr(VI) adsorbed at time t, (m^2·g^{-1}),

t – contact time, (min),

k_{id} – intra-particle diffusion rate constant (slope of the linear fit), (m^2·g^{-1} min$^{-1/2}$),

C –intercept related to the thickness of the boundary layer, (m^2·g^{-1}).

Equilibrium Tests

Adsorption isotherms are essential for understanding the interactions between adsorbent and adsorbate and thus for optimizing the efficiency of the adsorption process [9]. Equilibrium conditions were evaluated at initial Cr(VI) concentrations of 5, 10, 20, 30, 40, 60, 80, 100, and 150 mg L^{-1} and the contact time was 60 min. All experiments were performed at pH = 2.0 and were checked at the beginning and end of the experiment.

Although linear regression is commonly used to estimate model parameters, linearization can change dependent and independent variables, leading to error propagation. Therefore, nonlinear regression was used to improve the accuracy of parameter estimation because it avoids the disadvantages associated with linearization. It has been shown to be a more reliable and efficient method [71].

Four isotherm models were used to fit the data: the Redlich-Peterson (R-P), Freundlich, Langmuir and Dubinin-Radushkevich (D-R) models.

Langmuir isotherm assumes monolayer adsorption on a homogeneous surface with a finite number of identical sites and no interactions between adsorbed molecules. It is represented by the equation S5 [67]:

$$q_e = \frac{(q_{max} k_L c_e)}{(1 + k_L c_e)} \tag{S5}$$

where:

q_{max} – maximum adsorption capacity ($m^2 \cdot g^{-1}$),

k_L – Langmuir constant ($L \cdot mg^{-1}$),

c_e – equilibrium concentration of adsorbate ($mg \cdot L^{-1}$).

All other variables are the same as previously defined.

The Freundlich isotherm is one of the first empirical models used to describe equilibrium adsorption data and to characterize adsorption on heterogeneous surfaces. It is described by the equation S6 [67]:

$$q_e = k_F c_e^{1/n} \tag{S6}$$

where:

k_F – Freundlich constant [($m^2 \cdot g^{-1}$) ($L \cdot mg^{-1}$)$^{1/n}$],

n – adsorption intensity.

All other variables are as previously defined.

The R–P isotherm is an empirical model used for adsorption on heterogeneous surfaces. It includes both the Langmuir and Freundlich models and is expressed by the equation S7 [67]:

$$q_e = \frac{k_{RP} c_e}{1 + \alpha_{RP} c_e^g} \tag{S7}$$

where:

k_{RP} – Redlich–Peterson constants ($L \cdot g^{-1}$),

α_{RP} – Redlich–Peterson constant ($L \cdot mg^{-1}$),

g – exponent between 0 and 1.

All other variables are as previously defined.

The D-R isotherm was developed to account for the effect of porous structure on the adsorption process. It is represented by the equation **S**8 [67]:

$$q_e = q_{RD} e^{-k_{RD} \varepsilon^2} \tag{S8}$$

where:

q_{RD} – theoretical saturation capacity ($m^2 \cdot g^{-1}$),

k_{RD} – constant related to sorption energy ($mol^2 \cdot kJ^{-2}$),

ε – Polanyi potential.

Polanyi potential calculation (equation S9):

$$\varepsilon = \mathrm{R}T \ln(1 + \frac{1}{c_e}) \tag{S9}$$

where R is the gas constant and T the temperature in Kelvin.

Statistical Analysis and Model Fitting

Model parameters were estimated by nonlinear regression analysis. Unlike linearized forms of isotherm and kinetic models, which can distort the relationship between dependent and independent variables and propagate errors, nonlinear regression preserves the original mathematical structure of the models and typically provides more accurate and unbiased parameter estimates. For this reason, nonlinear fitting is considered a more robust approach for modelling adsorption processes [72].

All model fitting and statistical analyses were performed using OriginPro 2023 (OriginLab Corporation, USA). Parameters were obtained by nonlinear curve-fitting using the iterative optimization algorithms implemented in OriginPro, and goodness-of-fit was assessed primarily by the coefficient of determination (R^2). For completeness and transparency, standard errors of the fitted parameters and additional fit-quality metrics are reported, and residuals were examined to identify any systematic deviations.

Supplementary figures

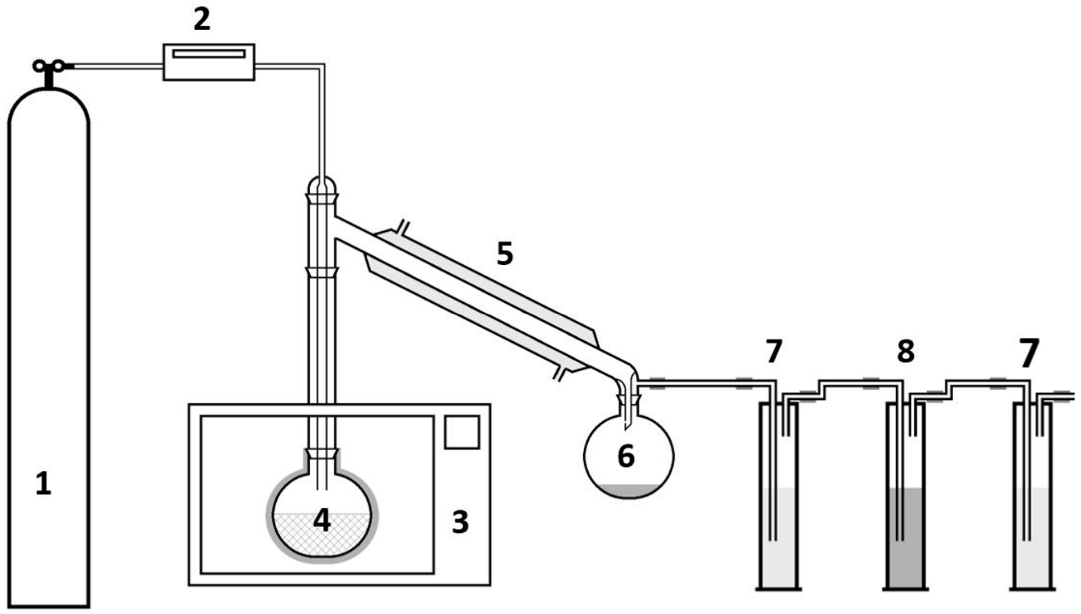

Figure S1: Diagram of microwave apparatus 1. Nitrogen pressure cylinder; 2. Nitrogen flow control; 3. Microwave oven; 4. Quartz reactor with a volume of 500 mL; 5. Cooler according to Liebig; 6. Condensation flask with a volume of 250 mL; 7. Washing flask with a volume of 250 mL with 70 mL of demineralized water; 8. Washing flask with a volume of 250 mL with 70 mL of Acetone (Penta, purity p.a.)

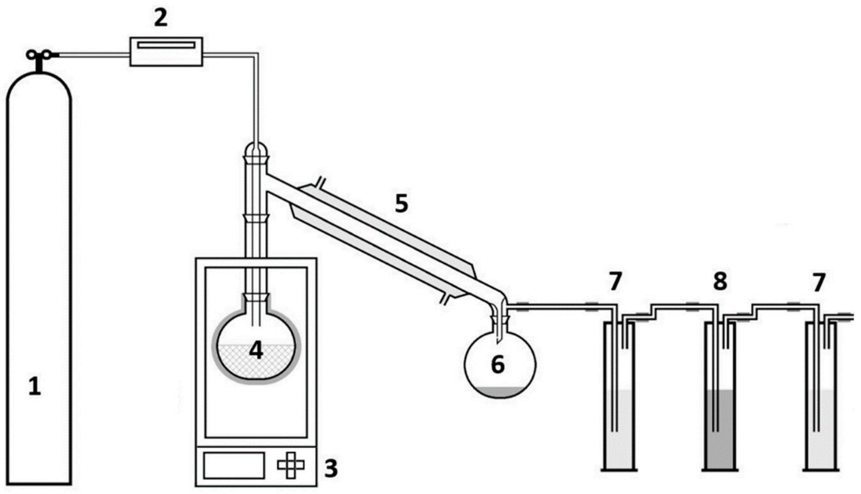

Figure S2: Schematic diagram of the apparatus for slow pyrolysis 1. Pressure bottle with nitrogen; 2. Nitrogen flow control; 3. Retort; 4. Quartz reactor with a volume of 500 mL; 5. Cooler according to Liebig; 6. Condensation flask with a volume of 250 mL; 7. Washing flask with a volume of 250 mL with 70 mL of demineralized water; 8. Washing flask with a volume of 250 mL with 70 mL of Acetone (Penta, purity p.a.).

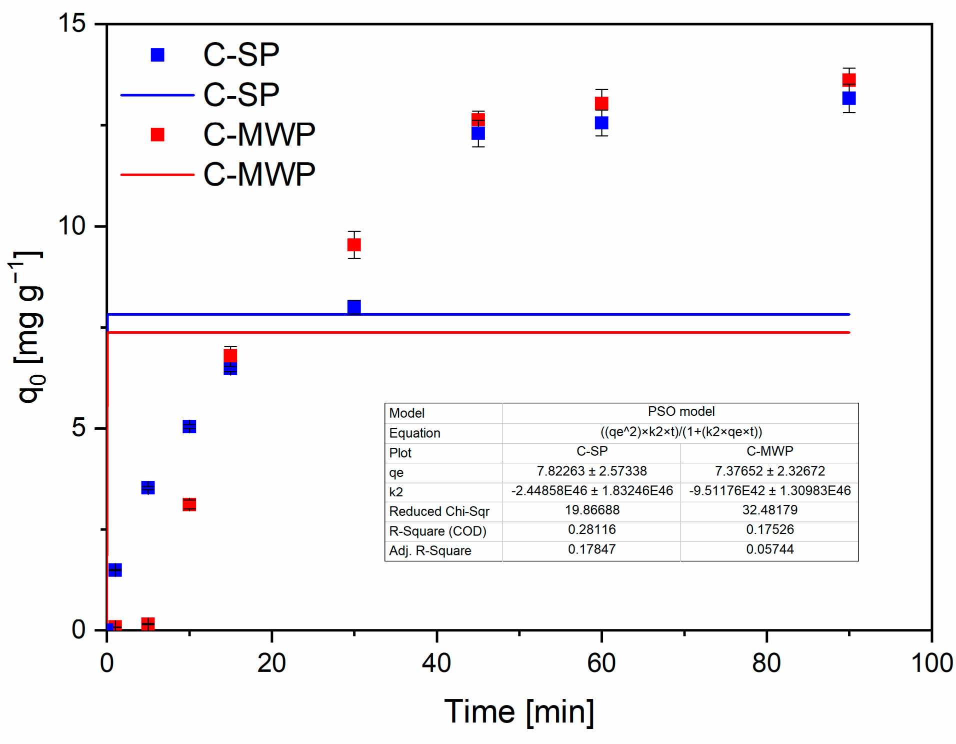

Figure S3. PSO fits and parameters (C-SP, C-MWP). Conditions: c_0 = 40 mg·L⁻¹; V = 20 mL; m = 0.05 g; pH = 2.0 (buffered); t = 0–90 min; T = 22 ±2 °C; rotation speed = 180 rpm. Data are presented as mean ± SD (n = 3). Error bars represent standard deviation. Fit method: nonlinear regression (OriginPro 2023).

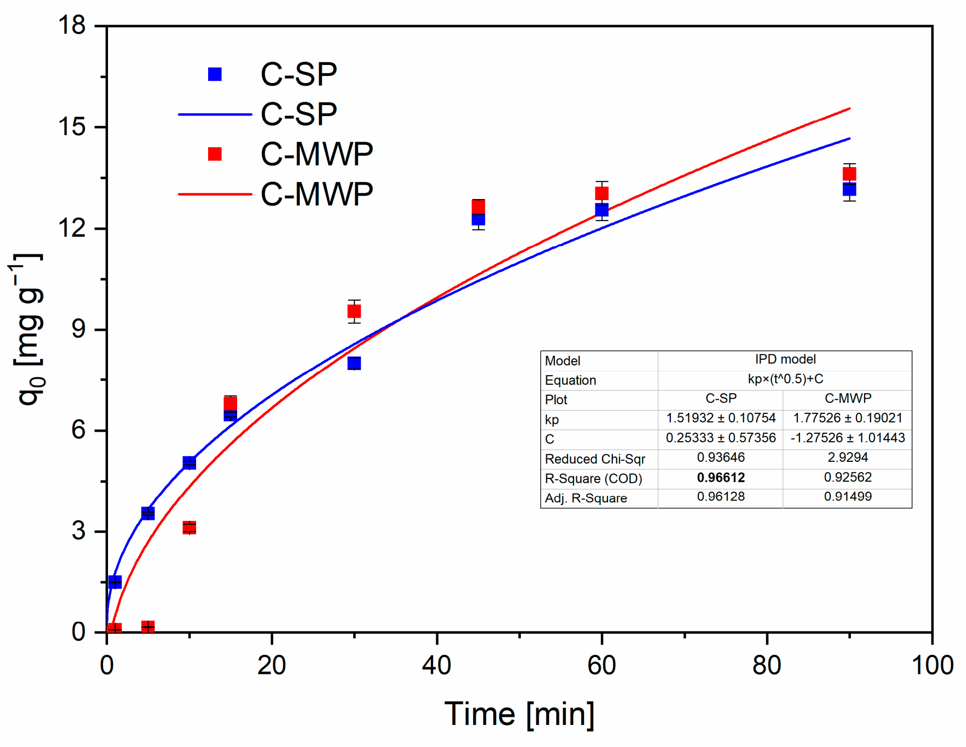

Figure S4: IPD fits and parameters (C-SP, C-MWP). Conditions: c_0 = 40 mg·L⁻¹; V = 20 mL; m = 0.05 g; pH = 2.0 (buffered); contact time = 0–90 min; T = 22 ± 2 °C; rotation speed = 180 rpm. Data are presented as mean ± SD (n = 3). Error bars represent standard deviation. Fit method: nonlinear regression (OriginPro 2023).

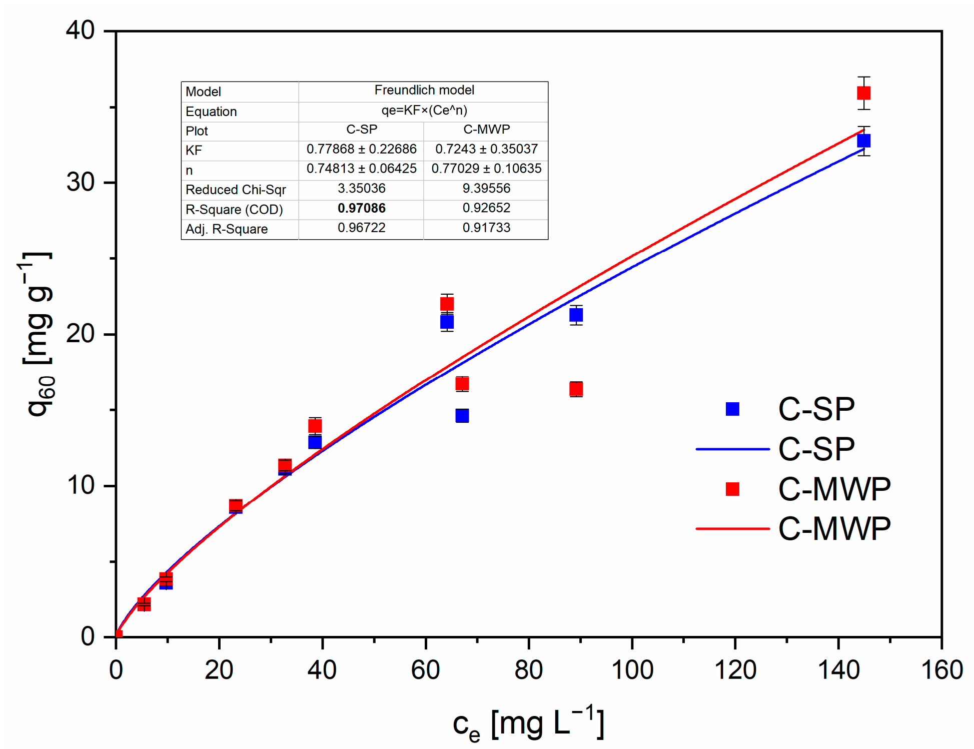

Figure S5: Freundlich adsorption isotherm for Cr (VI) removal by C-SP and C-MWP. Conditions: c_e = 5–150 mg·L^{-1}; V = 20 mL; m = 0.05 g; pH = 2.0 (buffered); contact time = 60 min; T = 22 ± 2 °C; rotation speed = 180 rpm. Data are presented as mean ± SD (n = 3). Error bars represent standard deviation. Fit method: nonlinear regression (OriginPro 2023).

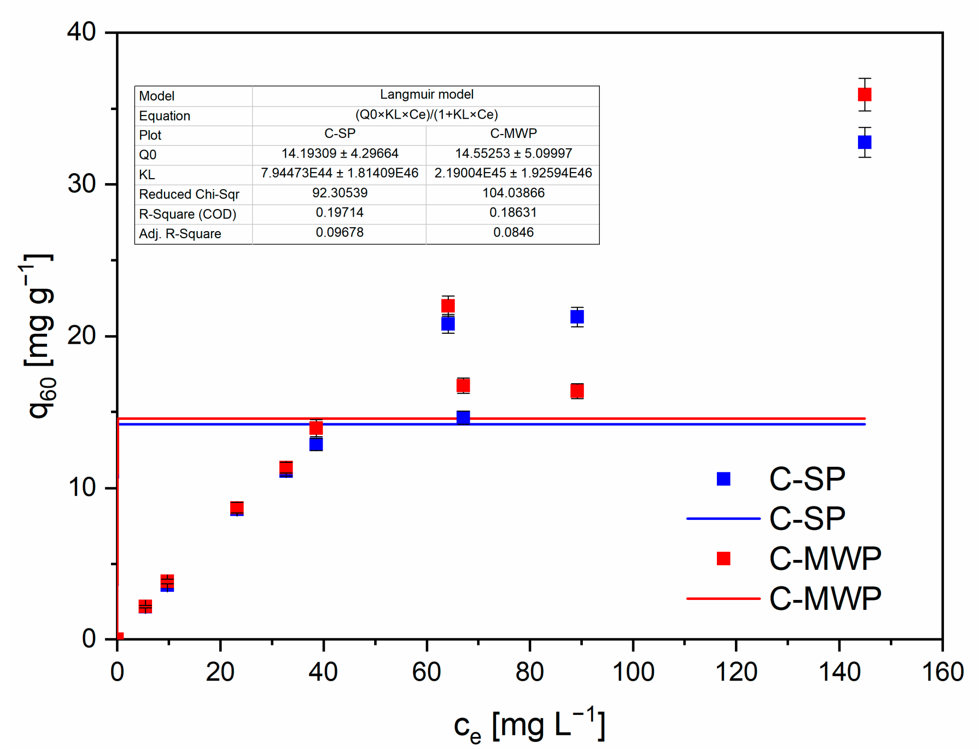

Figure S6: Langmuir adsorption isotherm for Cr (VI) removal by C-SP and C-MWP. Conditions: c_e = 5–150 mg·L^{-1}; V = 20 mL; m = 0.05 g; pH = 2.0 (buffered); contact time = 60 min; T = 22 ± 2 °C; rotation speed = 180 rpm. Data are presented as mean ± SD (n = 3). Error bars represent standard deviation. Fit method: nonlinear regression (OriginPro 2023).

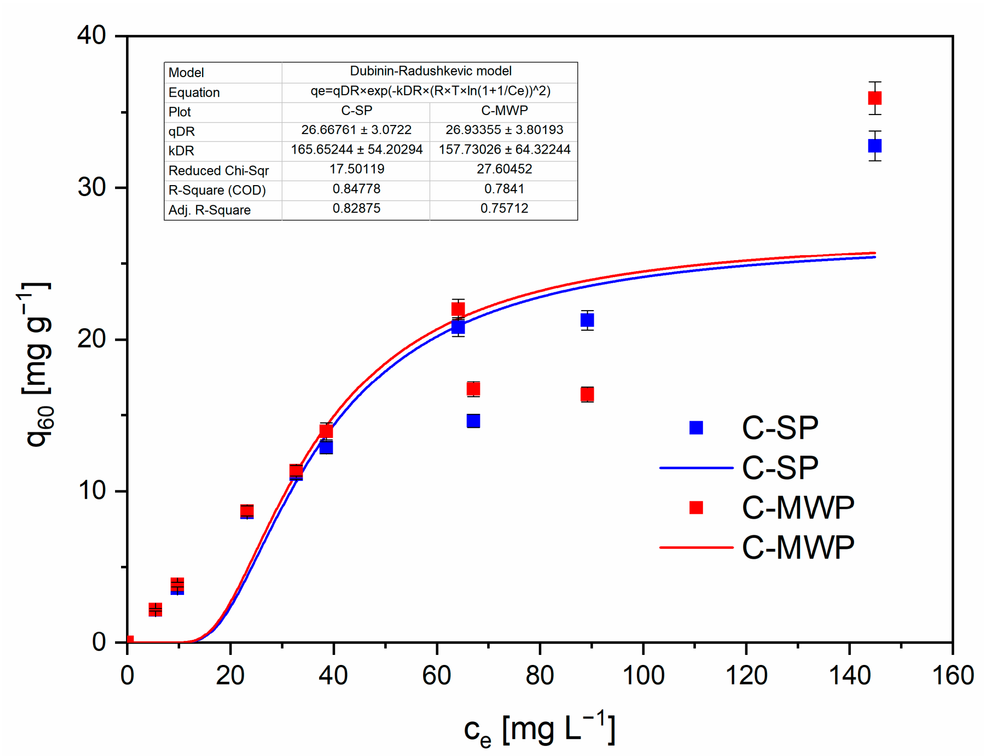

Figure S7: D-R adsorption isotherm for Cr (VI) removal by C-SP and C-MWP. Conditions: c_e = 5–150 mg·L^{-1}; V = 20 mL; m = 0.05 g; pH = 2.0 (buffered); contact time = 60 min; T = 22 ± 2 °C; rotation speed = 180 rpm. Data are presented as mean ± SD (n = 3). Error bars represent standard deviation. Fit method: nonlinear regression (OriginPro 2023).

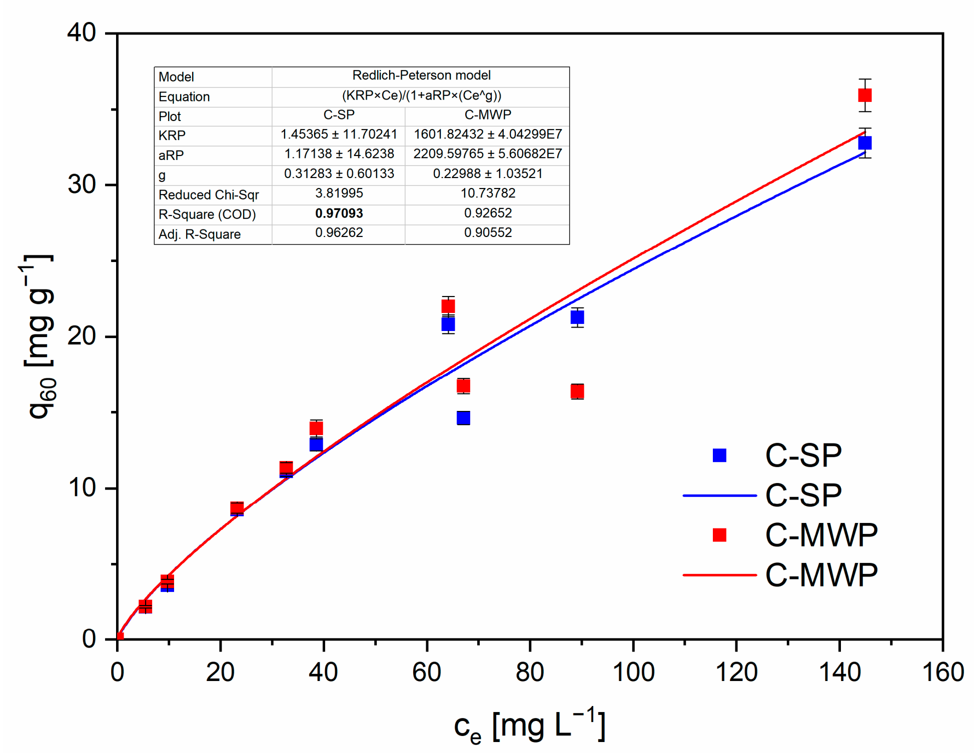

Figure S8: R-P adsorption isotherm for Cr (VI) removal by C-SP and C-MWP. Conditions: c_e = 5–150 mg·L^{-1}; V = 20 mL; m = 0.05 g; pH = 2.0 (buffered); contact time = 60 min; T = 22 ± 2 °C; rotation speed = 180 rpm. Data are presented as mean ± SD (n = 3). Error bars represent standard deviation. Fit method: nonlinear regression (OriginPro 2023).

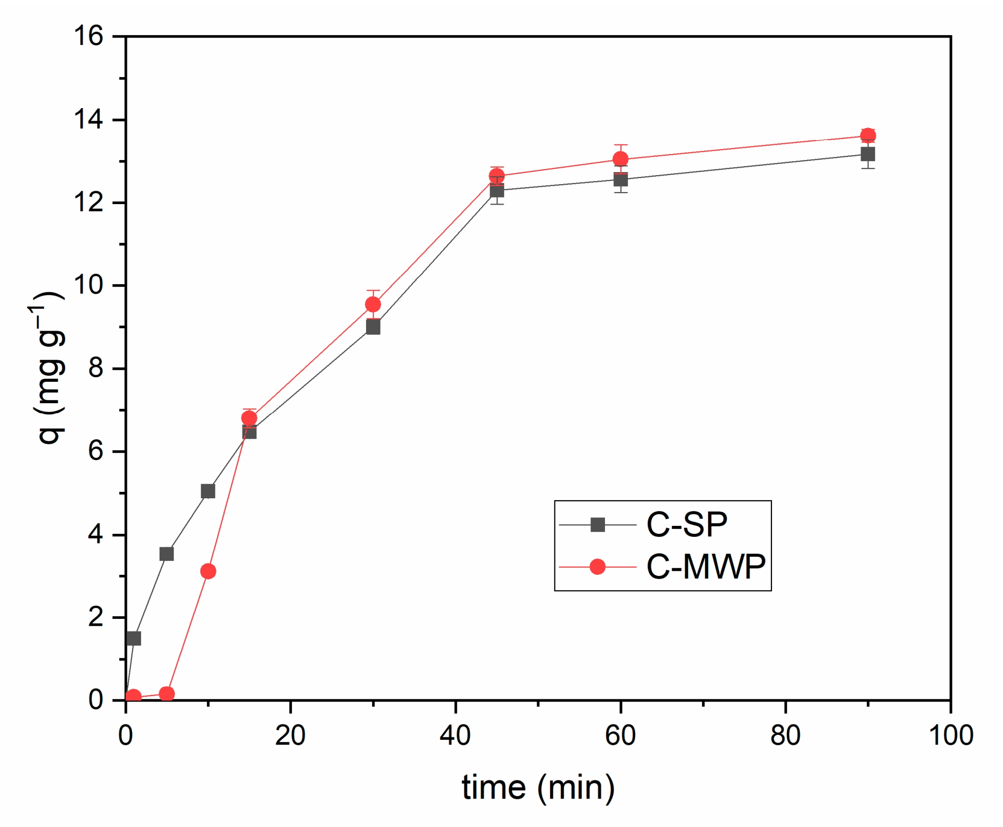

Figure S9: Representative kinetic curves (q_t vs time) for Cr (VI) removal by C-SP and C-MWP. Conditions: c_0 = 40 mg·L^{-1}; V = 20 mL; m = 0.05 g; pH = 2.0 (buffered); t = 0–90 min; T = 22 ±2 °C; rotation speed = 180 rpm. Data are presented as mean ± SD (n = 3). Error bars represent standard deviation. The vertical dashed line indicates the 60 min time used for equilibrium isotherm experiments.

Because the Langmuir model did not provide reliable/physical fits for all datasets, we report the experimentally observed maximum uptake ($q_{max,obs}$) as the primary comparative metric.

Table S1: Adsorption capacity of Cr(VI) by various biochars derived from lignocellulosic biomass (benchmarking focused on Cr(VI) to ensure comparability of pH, dose, and test conditions).

**Biochar source**	q_{max} **(mg·g⁻¹)**	**pH**	**Adsorbent dose**	**Pyrolysis conditions**	S_{BET} **(m²·g⁻¹)**	**Isotherm models**	**Kinetic models**	**Contact time min**	**Literature**
Cork powder	30.2	2	0.1 g / 50 mL	500 °C, 1 h, N_2	3.2	Langmuir, Freundlich	PSO	60	[57]
Almond shell + nZVI	26.63	6	0.08 g / 100 mL	600 °C, 2 h	21.28	Langmuir	PSO, Elovich	60	[58]
Pinecone	76.92	2	0.1 g / 50 mL	500 °C, 2 h	125	Langmuir, Freundlich	PSO	60	[59]
Zn-modified porous biochar	102.7	2	0.1 g Zn–biochar / 50 mL	450 °C, 1 h, N_2	21.28 (Zn–biochar)	Freundlich	PSO, PFO	2880	[60]
Tectona grandis sawdust	83.5	3	0.02 g / 50 mL	Hydrothermal carbonization 190 °C, 24 h	792	Langmuir	-	1440	[61]
Tectona grandis + $ZnCl_2$	127	3	0.02 g / 50 mL	800 °C, 4 h	1757	Langmuir	-	1440	[61]
Tectona grandis + K_2CO_3	103	3	0.02 g / 50 mL	800 °C, 4 h	1013	Langmuir	-	1440	[61]
Cork	32.7	2	0.1 g / 20 mL	600 °C, 1 h, N_2	175	Freundlich	PFO, Elovich, IPD	60	This work
Cork	35.9	2	0.1 g / 20 mL	MWP: 400 W	131	-	PFO, Elovich	60	This work

qmax,obs = experimentally observed maximum q_e from isotherm series (c_e = 5–150 mg·L⁻¹, pH 2.0, dose 2.5 g·L⁻¹, t_e = 60 min).

Calculation of apparent active-site density

Apparent active-site densities were estimated from the experimentally observed maximum uptake *qmax,obs* (mg·g⁻¹) measured at the highest equilibrium concentration (150 mg·L⁻¹). *qmax,obs* (mg·g⁻¹) was converted to mmol·g⁻¹ by dividing by the atomic mass of Cr (52.00 mg·mmol⁻¹), and then to the number of adsorbed atoms per gram by multiplying by Avogadro's number per mmol (6.022×10^{20} sites·mmol⁻¹). The number of sites·g⁻¹ was normalized by the specific surface area S_{BET} (m²·g⁻¹) and converted to sites·nm⁻² using 1 m² = 10^{18} n m². The working equation (constants rounded for clarity) is:

$$\text{site}\cdot\text{nm}^{-2} = \frac{q_{max}(\text{mg}\cdot\text{g}^{-1})}{52.00}\times\frac{602.2}{\text{BET}(\text{mg}\cdot\text{g}^{-1})}$$

Uncertainties were obtained by propagation of the relative errors of *qmax,obs* and S_{BET} (assuming independent errors). Reported values are apparent site densities under the 1:1 Cr : site assumption (one Cr atom per adsorption site). Important limitations are discussed below and in the main text: if Cr(VI) is reduced to Cr(III) with subsequent precipitation or formation of multinuclear complexes, or if parts of the BET area are inaccessible to the adsorbate, the calculated site densities are only approximate indicators for comparison between samples, not absolute measures of chemical binding-site counts. For precedents and discussion of this approach see e.g. [73, 74]. The values reported in Table S2 are apparent site densities under the 1:1 binding assumption.

Table S2: Apparent active-site densities estimated from *qmax,obs* and BET.

Sample	***qmax,obs* (mg·g⁻¹) (mean ± SD, *n*=3)**	**S_{BET} (m²·g⁻¹) (mean ± SD, *n*=2)**	**Apparent site density site·nm⁻²**
C-SP	32.7 ± 0.98	174.5 ± 0.11	2.2 ± 0.07
C-MWP	35.9 ± 1.07	130.9 ± 0.27	3.2 ± 0.10

Table S3: Stability controls for Cr(VI) (no adsorbent, measured at t = 45 min; mean ± SD, *n* = 3).

**Condition**	**c_0 control mg·L⁻¹**	**c_{45} control mg·L⁻¹**	**Mean change %**
pH 1.1	9.86±0.8	10.01±0.6	+1.52
pH 2.0	9.94±0.9	10.08±0.4	+1.41
pH 3.0	10.11±0.5	9.75±0.7	-3.56
pH 4.0	9.92±0.8	9.98±0.7	+0.61
pH 5.0	10.3±0.4	9.98±0.8	-3.11

Table S4: Full numerical fits, parameter standard errors and residuals for kinetics and isotherm models.

**C-SP**					
PFO	q_e mg·g⁻¹	k_1 min⁻¹	ReducedChi-Sqr	R^2	ARS
	13.41	0.045	0.41	0.985	0.983
PSO	q_e	k_2 g·mg⁻¹·min⁻¹	ReducedChi-Sqr	R^2	ARS
	7.82	-2.4×10^{46}	19.87	0.281	0.178
Elovich	α mg·g⁻¹·min⁻¹	β mg·g⁻¹	ReducedChi-Sqr	R^2	ARS
	1.05	0.22	0.44	0.984	0.982
IPD	k_{id} m²·g⁻¹ min⁻¹/²	C m²·g⁻¹	ReducedChi-Sqr	R^2	ARS
	1.53	0.31	0.90	0.968	0.963
Freundlich	k_F (mg·g⁻¹) (L·mg⁻¹)$^{1/n}$	n	ReducedChi-Sqr	R^2	ARS

	0.78	0.75		3.35	0.971	0.967
Langmuir	q_{max} $mg{\cdot}g^{-1}$	k_L $L{\cdot}mg^{-1}$		ReducedChi-Sqr	R^2	ARS
	14.19	$7.9{\times}10^{44}$		92.3	0.197	0.097
R-P model	k_{RP} $L{\cdot}g^{-1}$	α_{RP} L mg^{-1}	g	ReducedChi-Sqr	R^2	ARS
	1.45	1.17	0.31	3.82	0.971	0.962
D-R model	q_{RD} $mg{\cdot}g^{-1}$	k_{DR} $mol^2{\cdot}kJ^{-2}$		ReducedChi-Sqr	R^2	ARS
	26.67	165.7		17.50	0.85	0.829
**C-MWP**						
PFO	q_e $mg{\cdot}g^{-1}$	k_1 min^{-1}		ReducedChi-Sqr	R^2	ARS
	15.04	0.033		1.18	0.970	0.966
PSO	q_e mg g^{-1}	k_2 $g{\cdot}mg^{-1}{\cdot}min^{-1}$		ReducedChi-Sqr	R^2	ARS
	7.37	$-9.51{\times}10^{42}$		32.48	0.175	0.057
Elovich	α $mg{\cdot}g^{-1}{\cdot}min^{-1}$	β $mg{\cdot}g^{-1}$		ReducedChi-Sqr	R^2	ARS
	0.60	0.14		1.94	0.951	0.944
IPD	k_{id} $m^2{\cdot}g^{-1}$ $min^{-1/2}$	C $m^2{\cdot}g^{-1}$		ReducedChi-Sqr	R^2	ARS
	1.78	-1.25		2.93	0.926	0.915
Freundlich	k_F $(mg{\cdot}g^{-1})$ $(L{\cdot}mg^{-1})^{1/n}$	n		ReducedChi-Sqr	R^2	ARS
	0.72	0.77		9.40	0.92652	
Langmuir	q_{max} $mg{\cdot}g^{-1}$	k_L $L{\cdot}mg^{-1}$		ReducedChi-Sqr	R^2	ARS
	14.55	$2.19{\times}10^{45}$		104	0.186	0.085
R-P model	k_{RP} $L{\cdot}g^{-1}$	α_{RP} L mg^{-1}	g	ReducedChi-Sqr	R^2	ARS
	1,601	2,209	0.23	10.74	0.927	0.906
D-R model	q_{RD} $mg{\cdot}g^{-1}$	k_{RD} $mol^2{\cdot}kJ^{-2}$		ReducedChi-Sqr	R^2	ARS
	26.93	157.7		27.30	0.784	0.757

Table S5: Comparison of q at 60 min and 90 min (stability of apparent equilibrium).

Condition	q_{60} $mg{\cdot}g^{-1}$	q_{90} $mg{\cdot}g^{-1}$	Change (60→90) %
C-SP	12.66±0.45	13.17±0.40	4.0
C-MWP	13.04±0.30	13.62±0.32	4.4

Values show that q changes between 60 and 90 min were less than 4.5 % for all tested conditions, indicating that 60 min is an appropriate equilibrium contact time for the isotherm experiments.

Because the Langmuir model did not provide reliable/physical fits for all datasets, we report the experimentally observed maximum uptake ($q_{max,obs}$) as the primary comparative metric.

Table S6: Comparison of experimental adsorption capacity $q_{e,exp}$ (measured at t = 60 min; mean ± SD, n = 3) with model-predicted $q_{PFO}(60)$ and $q_{elovich}(60)$. Absolute percent deviations |%dev|| are reported. Full fit parameters are listed in Table S4.

Example	$q_{e,exp}$ mg·g⁻¹	SD mg·g⁻¹	q_{PFO} (60) mg·g⁻¹	|%dev| PFO	$q_{elovich}$ (60) mg·g⁻¹	|%dev| Elovich
C-SP pH 2	12.66	±0.45	12.51	1.2	12.27	3.1
C-MWP pH 2	13.04	±0.30	12.96	0.6	12.85	1.5

Note:

The calculated values were obtained according to the following equations:

$$q_{PFO}(t_{eq}) = q_{e,fit}(1 - e^{-k_1 t_{eq}})$$

$$q_{Elovich}(t_{eq}) = \frac{1}{\beta}\ln(1 + \alpha\beta t_{eq})$$

$$\%\ deviation = 100 \times \frac{q_{model}(t_{eq}) - q_{e,exp}}{q_{e,exp}}$$

where $q_{e,fit}$ and k_1 are the parameters of the PFO model, and α and β are the parameters of the Elovich model. Interpretation: <5 % = very good agreement; 5–15 % = moderate deviation; >15 % = significant deviation.

Table S7: XRF elemental composition of cork-derived biochars (C-SP, C-MWP).

Specified element		w/w [%]		Specified element		w/w [%]	
		C-SP	C-MWP			C-SP	C-MWP
Na	Sodium	< 0.01	0.012	Y	Yttrium	0.001	0.001
Mg	Magnesium	0.125	0.130	Zr	Zirconium	0.022	0.023
Al	Aluminum	0.056	0.060	Nb	Niobium	0.0005	0.000
Si	Silicon	0.254	0.260	Mo	Molybdenum	0.001	0.001
P	Phosphorus	0.852	0.845	Ag	Silver	< 0.0002	< 0.0002
S	Sulfur	0.001	0.002	Cd	Cadmium	0.0001	0.0001
Cl	Chlorine	0.025	0.028	Sn	Tin	0.0001	0.0001
K	Potassium	0.0005	0.001	Sb	Antimony	< 0.0003	< 0.0003
Ca	Calcium	1.028	1.035	Te	Tellurium	< 0.0003	< 0.0003
Ti	Titanium	0.131	0.129	I	Iodine	< 0.0003	< 0.0003
V	Vanadium	0.002	0.003	Cs	Cesium	< 0.0004	< 0.0004
Cr	Chromium	0.005	0.006	Ba	Barium	0.148	0.150
Mn	Manganese	0.125	0.120	La	Lanthanum	0.011	0.010
Fe	Iron	0.002	0.003	Ce	Cerium	0.019	0.019
Co	Cobalt	< 0.0003	< 0.0003	Pr	Praseodymium	0.005	0.005
Ni	Nickel	0.011	0.010	Nd	Neodymium	0.014	0.014
Cu	Copper	0.007	0.008	Hf	Hafnium	0.0005	0.0005
Zn	Zinc	0.029	0.030	Ta	Tantalum	0.008	0.008
Ga	Gallium	0.001	0.001	W	Tungsten	< 0.00013	< 0.00013
Ge	Germanium	0.0001	0.0001	Hg	Mercury	< 0.00007	< 0.00007
As	Arsenic	0.0003	0.0003	Tl	Thallium	0.0002	0.0002
Se	Selenium	0.0002	0.0002	Pb	Lead	0.001	0.001

Br	Bromine	0.004	0.004	Bi	Bismuth	< 0.0001	< 0.0001
Rb	Rubidium	0.018	0.018	Th	Thorium	0.001	0.001
Sr	Strontium	0.087	0.089	U	Uranium	< 0.0001	< 0.0001

References

63. ASTM D7582; Standard Test Methods for Proximate Analysis of Coal and Coke by Macro Thermogravimetric Analysis. ASTM International: West Conshohocken, PA, USA, 2015
64. Sing, K.S.W. Reporting Physisorption Data for Gas/Solid Systems with Special Reference to the Determination of Surfac$_e$ Area and Porosity (Recommendations 1984). Pure Appl. Chem. 1985, 57, 603–619, doi:10.1351/pac198557040603.
65. Donohue, M.D.; Aranovich, G.L. Classification of Gibbs Adsorption Isotherms. Adv. Colloid Interfac$_e$ Sci. 1998, 76–77, 137–152, doi:10.1016/S0001-8686(98)00044-X.
66. Hasan, S.H.; Singh, K.K.; Prakash, O.; Talat, M.; Ho, Y.S. Removal of Cr(VI) from Aqueous Solutions Using Agricultural Waste 'Maize Bran.' J. Hazard. Mater. 2008, 152, 356–365, doi:10.1016/j.jhazmat.2007.07.006.
67. Lang, J.; Bednárek, J.; Ritz, M.; Kormunda, M.; Zelenka, T.; Vaštyl, M.; Gavlová, A.; Kolská, Z.; Férová, M. Microporous Carbonac$_e$ous Adsorbent Prepared from a Pyrolyzed Polymer. Mater. Adv. 2024, 5, 6458–6468, doi:10.1039/D4MA00141A.
68. Ho, Y.S.; McKay, G. The Sorption of Lead(II) Ions on Peat. Water Res. 1999, 33, 578–584, doi:10.1016/S0043-1354(98)00207-3.
69. Hu, X.; Wang, J.; Liu, Y.; Li, X.; Zeng, G.; Bao, Z.; Zeng, X.; Chen, A.; Long, F. Adsorption of Chromium (VI) by Ethylenediamine-Modified Cross-Linked Magnetic Chitosan Resin: Isotherms, Kinetics and Thermodynamics. J. Hazard. Mater. 2011, 185, 306–314, doi:10.1016/j.jhazmat.2010.09.034.
70. Edet, U.A.; Ifelebuegu, A.O. Kinetics, Isotherms, and Thermodynamic Modeling of the Adsorption of Phosphates from Model Wastewater Using Recycled Brick Waste. Proc$_e$sses 2020, 8, 665, doi:10.3390/pr8060665.
71. Chen, X.; Hossain, M.F.; Duan, C.; Lu, J.; Tsang, Y.F.; Islam, M.S.; Zhou, Y. Isotherm Models for Adsorption of Heavy Metals from Water - A Review. Chemosphere 2022, 307, 135545, doi:10.1016/j.chemosphere.2022.135545.
72. Wang, J.; Guo, X. Adsorption Isotherm Models: Classification, Physical Meaning, Application and Solving Method. Chemosphere 2020, 258, 127279, doi:10.1016/j.chemosphere.2020.127279.
73. Frini-Srasra, N.; Srasra, E. Adsorption of Quinalizarin from Non Aqueous Solution onto Acid Activated Palygorskite. Surf. Eng. Appl. Electrochem. 2009, 45, 306–311, doi:10.3103/S1068375509040103.
74. Bae, G.; Kim, H.; Choi, H.; Jeong, P.; Kim, D.H.; Kwon, H.C.; Lee, K.-S.; Choi, M.; Oh, H.-S.; Jaouen, F.; et al. Quantification of Active Site Density and Turnover Frequency: From Single-Atom Metal to Nanoparticle Electrocatalysts. JACS Au 2021, 1, 586–597, doi:10.1021/jacsau.1c00074.
